# Antibody-protein interactions: benchmark datasets and prediction tools evaluation

**DOI:** 10.1186/1472-6807-7-64

**Published:** 2007-10-02

**Authors:** Julia V Ponomarenko, Philip E Bourne

**Affiliations:** 1 San Diego Supercomputer Center, University of California, San Diego, 9500 Gilman Drive, La Jolla, California 92093, USA; 2 Skaggs School of Pharmacy and Pharmaceutical Sciences, University of California, San Diego, 9500 Gilman Drive, La Jolla, California 92093, USA

## Abstract

**Background:**

The ability to predict antibody binding sites (aka antigenic determinants or B-cell epitopes) for a given protein is a precursor to new vaccine design and diagnostics. Among the various methods of B-cell epitope identification X-ray crystallography is one of the most reliable methods. Using these experimental data computational methods exist for B-cell epitope prediction. As the number of structures of antibody-protein complexes grows, further interest in prediction methods using 3D structure is anticipated. This work aims to establish a benchmark for 3D structure-based epitope prediction methods.

**Results:**

Two B-cell epitope benchmark datasets inferred from the 3D structures of antibody-protein complexes were defined. The first is a dataset of 62 representative 3D structures of protein antigens with inferred structural epitopes. The second is a dataset of 82 structures of antibody-protein complexes containing different structural epitopes. Using these datasets, eight web-servers developed for antibody and protein binding sites prediction have been evaluated. In no method did performance exceed a 40% precision and 46% recall. The values of the area under the receiver operating characteristic curve for the evaluated methods were about 0.6 for ConSurf, DiscoTope, and PPI-PRED methods and above 0.65 but not exceeding 0.70 for protein-protein docking methods when the best of the top ten models for the bound docking were considered; the remaining methods performed close to random. The benchmark datasets are included as a supplement to this paper.

**Conclusion:**

It may be possible to improve epitope prediction methods through training on datasets which include only immune epitopes and through utilizing more features characterizing epitopes, for example, the evolutionary conservation score. Notwithstanding, overall poor performance may reflect the generality of antigenicity and hence the inability to decipher B-cell epitopes as an intrinsic feature of the protein. It is an open question as to whether ultimately discriminatory features can be found.

## Background

A B-cell epitope is defined as a part of a protein antigen recognized by either a particular antibody molecule or a particular B-cell receptor of the immune system [1]. The main objective of B-cell epitope prediction is to facilitate the design of a short peptide or other molecule that can be synthesized and used instead of the antigen, which in the case of a pathogenic virus or bacteria, may be harmful to a researcher or experimental animal [2]. A  B-cell epitope may be continuous, that is, a short contiguous stretch of amino acid residues, or discontinuous, comprising atoms from distant residues but close in three-dimensional space and on the surface of the protein.

Synthetic peptides mimicking epitopes, as well as anti-peptide antibodies, have many applications in the diagnosis of various human diseases [3-7]. Also, the attempts have been made to develop peptide-based synthetic prophylactic vaccines for various infections, as well as therapeutic vaccines for chronic infections and noninfectious diseases, including autoimmune diseases, neurological disorders, allergies, and cancers [8-10]. The immunoinformatics software and databases developed to facilitate vaccine design have previously been reviewed [11,12].

During the last 25 years B-cell epitope prediction methods have focused primarily on continuous epitopes. They were mostly sequence-dependent methods based upon various amino acid properties, such as hydrophilicity [13], solvent accessibility [14], secondary structure [15-18], and others. Recently, several methods using machine learning approaches have been introduced that apply hidden Markov models (HMM) [19], artificial neural networks (ANN) [20], support vector machine (SVM) [21], and other techniques [22,23]. Recent assessments of continuous epitope prediction methods demonstrate that "single-scale amino acid propensity profiles cannot be used to predict epitope location reliably" [24] and that "the combination of scales and experimentation with several machine learning algorithms showed little improvement over single scale-based methods" [25].

As crystallographic studies of antibody-protein complexes have shown, most B-cell epitopes are discontinuous. In 1984, the first attempts at epitope prediction based on 3D protein structure was made for a few proteins for which continuous epitopes were known [26-28]. Subsequently, Thornton and colleagues [29] proposed a method to locate potential discontinuous epitopes based on a protrusion of protein regions from the protein's globular surface. However, until the first X-ray structure of an antibody-protein complex was solved in 1986 [30], protein structural data were mostly used for prediction of continuous rather than discontinuous epitopes.

In cases where the three-dimensional structure of the protein or its homologue is known, a discontinuous epitope can be derived from functional assays by mapping onto the protein structure residues involved in antibody recognition [31]. However, an epitope identified using an immunoassay may be an artefact of measuring cross-reactivity of antibodies due to the presence of denatured or degraded proteins [32,33], or due to conformational changes in the protein caused by residue substitutions that may even lead to protein mis-folding [34]. Therefore, structural methods, particularly X-ray crystallography of antibody-antigen complexes, generally identify B-cell epitopes more reliably than functional assays [35].

B-cell epitopes can be thought of in a structural and functional sense. Structural epitopes (also called antigenic determinants) are defined by a set of residues or atoms in the protein antigen contacting antibody residues or atoms [33,36]. In contrast, a functional epitope consists of antigen residues that contribute significantly to antibody binding [36,37]. Functional epitopes are determined through functional assays (e.g., alanine scanning mutagenesis) or calculated theoretically using known structures of antibody-protein complexes [38,39]. Thus, functional and structural epitopes are not necessary the same. Functional epitopes in proteins are usually smaller than structural epitopes; only three to five residues of the structural epitope contribute significantly to the antibody-antigen binding energy [40]. This work focuses on structural epitopes inferred from known 3D structures of antibody-protein complexes available in the Protein Data Bank (PDB) [41].

Antibody-protein complexes can be categorized as intermediate transient non-obligate protein-protein complexes [40,42]. Non-obligate complexes, implying that individual components can be found on their own *in vivo*, are classified as either permanent or transient depending on their stability under particular physiological and environmental conditions [43]. For example, many enzyme-inhibitor complexes are permanent non-obligate complexes. Transient non-obligate complexes range from weak (e.g., electron transport complexes), to intermediate (e.g., signal transduction complexes), and to strong (e.g., bovine G protein forming a stable trimer upon GDP binding) [44]. Most antibodies demonstrate intermediate affinity for their specific antigens [45]. Based on this classification, general methods for the prediction of intermediate transient non-obligate protein-protein interactions have been applied to the prediction of structural epitopes [40,42]. For example, Jones and Thornton, using their method for predicting protein-protein binding sites [46], successfully predicted B-cell epitopes on the surface of the β-subunit of human chorionic gonadotropin (βhCG) [47].

Since the number of available structures of antibody-protein complexes remains limited, thus far only a few methods, CEP (Conformational Epitope Prediction) [48] and DiscoTope [49], for B-cell epitope prediction using a protein of a given three-dimensional structure have been developed. In the near future, with growth in the number of available structures of antibody-protein complexes, extensive development in this area is expected. Existing and new methods for epitope prediction demand a benchmark which will set the standard for the future comparison of methods. To facilitate the further development of this standard, we have developed B-cell epitope benchmark datasets inferred from existing 3D structures of antibody-protein complexes. Further, using the benchmark datasets, we evaluated CEP, DiscoTope, and six recently developed publicly available web-servers for generalized protein-protein binding site prediction using various approaches: protein-protein docking (ClusPro [50], DOT [51] and PatchDock [52]); structure-based methods applying different principals and trained on different datasets (PPI-PRED [53], PIER [54] and ProMate [55]), and residue conservation (ConSurf [56]).

## Results and discussion

### Structural epitope definition

Three definitions of an epitope inferred from the X-ray structures of antibody-protein complexes were considered: (1) The epitope consists of protein antigen residues in which any atom of the residue looses more than 1Å^2 ^of accessible surface area (ASA) upon antibody binding. ASA was calculated using the program NACCESS [57]; (2) The epitope consists of protein antigen residues in which any atom of the epitope residue is separated from any antibody atom by a distance ≤ 4Å; (3) The epitope consists of protein antigen residues in which any atom of the epitope residue is separated from any antibody atom by a distance ≤ 5Å. These three definitions were used for two reasons. First, the methods evaluated in this work use one of these three definitions, second, we wished to study how the epitope definition influenced the results.

Results (not shown) indicated that the structural epitope definition did not influence the outcome. Hence, unless otherwise specified, results are based on the second epitope definition.

### Construction of the benchmark datasets

Two benchmark datasets were derived from the 3D structures of antibody-protein complexes available from the PDB [41]:

• Dataset #1 – Representative 3D structures of protein antigens with structural epitopes inferred from 3D structures of antibody-protein complexes. This dataset is intended for the study of the antigenic properties of proteins as well as for development and evaluation of the methods based on protein structure alone, or protein-protein unbound docking methods, that is, if the structure of the antibody is known or can be modeled. Here this dataset was used for the evaluation of scale-based methods (DiscoTope, PIER, ProMate and ConSurf). The dataset contains 62 antigens, 52 of which are one-chain antigen proteins.

• Dataset #2 – Representative 3D structures of antibody-protein complexes presenting different epitopes. This dataset is useful for the study of the properties of individual epitopes as well as for development and evaluation of protein-protein bound docking methods. Since the current work attempts to compare the methods of different types, including protein-protein docking methods, this dataset was used to compare the performance of all methods to each other. The dataset contains 70 structures of proteins in complexes with two-chain antibodies and 12 structures of proteins in complexes with one-chain antibodies.

**Figure 1:**
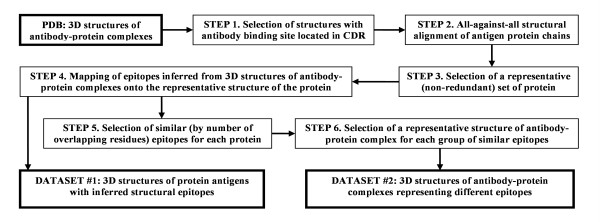
Flowchart for building benchmark datasets.

The flowchart describing the construction of the benchmark datasets is shown in Figure 1. Steps from 1 to 4 relate to dataset #1; steps 1–6 relate to dataset #2.

*Step 1 *– crystal structures of protein antigens of length ≥30 amino acids at a resolution ≤ 4Å in complex with antibody fragments containing variable regions (Fab, VHH, Fv, or scFv fragments) were collected from the Protein Data Bank (PDB) [41]. Structures in which the antibody binds antigen but involves no CDR residues have been excluded from the analysis; there were four such structures [PDB: 1MHH, 1HEZ, 1DEE, 1IGC]. If a structure contained several complexes in one asymmetric unit and there was no structural difference observed between these complexes, only one complex was selected. In this way 166 structures containing 187 antibody-protein complexes were selected: 24 complexes were formed by one-chain antibody fragments and 163 complexes by two-chain antibody fragments.

**Figure 2:**
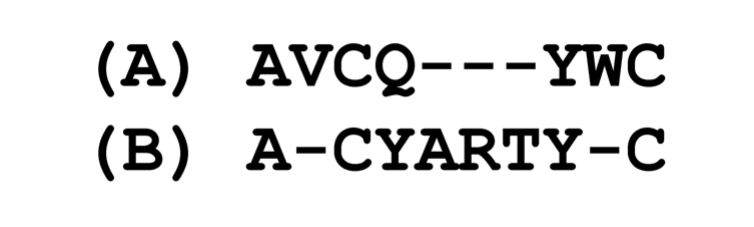
**Hypothetical example of the structural alignment of proteins (A) (sequence AVCQYWC) and (B) (sequence ACYARTYC)**. Number of residue-residue matches = 5, number of residue-residue matches relative to the length the longest chain = 63% (5/8), sequence identity = 80% (4/5).

*Step 2 *– all antigen protein chains were structurally aligned to one another using the CE algorithm [58]. Two protein chains were considered similar if all the following conditions applied: (i) rmsd ≤3Å, (ii) z-score ≥4.0, (iii) number of residue-residue matches relative to the length of the longest chain ≥80%, (iv) sequence identity in the structural alignment (not considering gaps) ≥80%. The z-score takes into account overall structural similarity and number of gapped positions. Two protein molecules were considered similar if each chain in one protein had a similar chain in another protein. Figure 2 demonstrates how the last two parameters, number of matches and sequence identity in the structural alignment, are defined.

The structural alignment rather than sequence alignment was used because protein structure is more conserved than sequence, and there can be expected regions in proteins with low sequence similarity that cannot be aligned by sequence alone. The structural alignment also avoids considering two proteins as similar if they have similar sequences but different structures (possible over short regions). The threshold values were chosen empirically based on previous experience working with the CE algorithm. As a result, the chosen threshold values separated human and bird lysozymes (61% sequence identity) and neuraminidases of different influenza virus strains, H3N2 and H11N9 (47% sequence identity).

*Step 3 *– 35 proteins were orphans represented by only one 3D structure. Of the remaining 27 proteins represented by more than one 3D structure, the structure with the best resolution was selected as the representative structure. The final representative dataset contained 62 antigens [see Additional file 1], 52 of which were one-chain antigen proteins.

**Figure 3:**
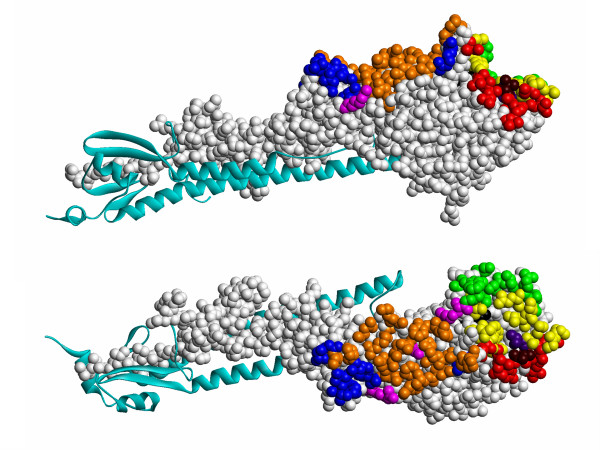
**Two orthogonal views of a representative structure, influenza A virus hemagglutinin HA1 chain [PDB:1EO8]**. Chain A is shown in light gray upon which are mapped epitope residues inferred from six protein structures in complexes with antibody fragments: HC45 Fab [PDB:1QFU] (blue), BH151 Fab [PDB:1EO8] (magenta), HC63 Fab [PDB:1KEN] (green), HC19 Fab [PDB:2VIR, 2VIS, 2VIT] (red). The hemagglutinin HA2 chain is shown in cyan. Residues common to HC45 and BH151 epitopes are shown in orange; residues common to HC63 and HC19 epitopes are shown in yellow; residue Tyr98 which is a part of HC19 epitope inferred from structure 2VIR but not from 2VIS and 2VIT structures is shown in black; The HC19 epitope residue Thr131 which is mutated to Ile in the 2VIS structure is shown in dark red. The HC19 epitope residue Thr155 which is mutated to Ile in 2VIT structure is shown in violet.

*Step 4 *– for each protein, epitopes inferred from the 3D structures of antibody-protein complexes were mapped onto the representative structure of the protein. First, epitope residues were calculated for each complex structure using one of the aforementioned epitope definitions. Second, epitope residues defined for the represented structures were mapped onto the representative structure based on the structure alignments. For example, the hemagglutinin HA1 chain of influenza A virus was represented by six 3D structures of the protein in complexes with Fab fragments of antibodies HC45 [PDB:1QFU], BH151 [PDB:1EO8], HC63 [PDB:1KEN], and HC19 [PDB:2VIR, 2VIS, 2VIT]. Figure 3 illustrates a representative structure [PDB:1EO8] of hemagglutinin HA1 upon which epitopes are mapped having been inferred from six complex structures. In this way, epitopes inferred from 187 structures of antibody-protein complexes were mapped onto the 62 representative protein structures. The resulting dataset is denoted dataset #1. Data on mapped epitope residues are available upon request.

*Step 5 *– to study the properties of individual epitopes and their prediction a dataset of representative epitopes, dataset #2 derived from 3D structures of antibody-protein complexes defining different epitopes was constructed. An important question to consider is how to define individual epitopes yet avoid bias by over-presentation of particular epitopes? For example (Fig. 3), while HC45 (blue) and BH151 (magenta) epitopes overlap, neither HC63 (green) nor HC19 (red) epitopes overlap, they are separated on the protein surface. Nevertheless, HC45 and BH151 epitopes share residues (orange in Fig. 3), as do HC63 and HC19 epitopes (yellow in Fig. 3). Are HC45 and BH151 epitopes similar or different? This question is answered by considering the degree of overlap.

Two epitopes are deemed similar if, in addition to the aforementioned criteria for epitope definition, they belong to similar protein chains and have >75% residues in common for both epitopes. A cut-off value of 75% for epitope similarity was chosen empirically. Thus, the HC45 and BH151 epitopes on influenza A virus hemagglutinin HA1 (Fig. 3) share 14 residues, that make up 74% and 93% of the size of HC45 and BH151 epitopes, respectively. A cut-off on epitope overlap of less than 75% would define HC45 and BH151 epitopes as similar even though they are known to be different. HC45 and BH151 are antibodies from different germ-lines with variable domains sharing only 56% sequence similarity, their H3 CDR regions adopt distinct conformations and these antibodies are tolerant to different mutations in hemagglutinin [59]. Another example, X5 and 17B epitopes of gp120 share 75% of their residues yet X5 and 17B antibodies are from different genes [60]. A cut-off value for epitope similarity equal to or less than 75% would erroneously define X5 and 17B epitopes as similar. Conversely, a cut-off value of 80% would make epitopes inferred from different structures of the same antibody-protein complex dissimilar. For example, the H57 epitope of T cell receptor N15 is inferred from two complex structures of a single crystal asymmetric unit ([PDB:1NFD], complexes (D)-(HG) and (B)-(FE), where the letters denote protein chain identifiers) would be dissimilar.

Given a 75% empirical cut-off for epitope similarity, epitopes inferred from structures of complexes with two-chain antibody fragments were divided into 44 singletons and 26 groups; epitopes inferred from structures of complexes with one-chain antibody fragments were divided into ten singletons and two groups.

*Step 6 *– for each group of similar epitopes, the representative 3D structure of the antibody-protein complex was selected based upon the following preferences. First, the structure with no or a minimal number of heteroatoms (excluding water) and other protein chains in the interface (i.e., separated from any atoms of both antigen and antibody by ≤4Å distance) was preferred. Second, preference was given to the structure with the largest epitope, i.e., maximum number of epitope residues. Third, the structure with the best resolution ≤2.5Å was preferred. Dataset #2 of representative structures of antibody-protein complexes (representative epitopes) consisted of 70 structures of proteins in complexes with two-chain antibody fragments and 12 structures of proteins in complexes with one-chain antibody fragments.

### Web-servers performance evaluation

Using the benchmark datasets introduced above we evaluated eight recently-developed and publicly available web-servers. The servers use different methods yet all have the goal of predicting either B-cell epitopes, or more generally protein-protein binding sites. The servers are listed in Table 1. Any reference in the text to the method actually means the server which implements that method, e.g., the DOT method running on the ClusPro server is called ClusPro(DOT).

**Table 1:** Servers evaluated in this work

Server name	Method type	Training dataset	Reference
CEP (Conformational Epitope Prediction)	Discontinuous epitope prediction based on residue solvent accessibility and spatial distribution.	No training set.	[48]
DiscoTope	Discontinuous epitope prediction based on amino acid statistics, residue solvent accessibility and spatial distribution.	75 structures of antibody-antigen complexes.	[49]
ProMate	Protein-protein binding interface prediction based on significant structural and sequence interface properties.	Manually curated; 57 protein involved in heterodimeric transient interactions (excluding antigen-antibody complexes).	[55]
PIER (Protein IntErface Recognition)	Protein-protein binding interface prediction based on local statistical properties of the protein surface derived at the level of atomic groups.	490 homodimeric, 62 heterodimeric and 196 transient interfaces (excluding antigen-antibody complexes).	[54]
PPI-PRED (Protein-Protein Interface Prediction)	Protein-protein binding interface prediction based on significant structural and sequence interface properties.	Manually curated; 180 proteins from 149 complexes both obligate (114) and transient (66).	[53]
ConSurf	Mapping of phylogenetic information (sequence conservation grades) on to the surface of proteins with known 3D structure.	No training set.	[56]
ClusPro (DOT program)	Rigid-body protein-protein docking based on the Fast-Fourier Transform correlation approach.	No training set.	[50] [51]
PatchDock	Rigid-body protein-protein docking based on local shape feature matching.	No training set.	[52]

The methods fall into two categories:

• *Scale-based methods *– each protein residue is assigned a value reflecting the probability of that residue being part of the protein interface or epitope. DiscoTope, PIER, ProMate and ConSurf fall into this category.

• *Patch prediction and protein-protein docking methods *– each protein residue is predicted to be part of a surface patch of residues defining the protein interface or epitope. DiscoTope, ProMate, CEP, PPI-PRED, ClusPro(DOT), and PatchDock fall into this category.

Two methods, DiscoTope and ProMate, fall into both categories since they predict patches and assign score values to each protein residue.

The evaluation of the methods was performed as follows. First, the scale-based methods were analyzed on how well the residue score values discriminate epitope versus non-epitope residues using dataset #1. Further, performance of all methods was evaluated on their ability to recognize representative epitopes from dataset #2. The first step is obviously not essential; it was performed as an example of the application of dataset #1 that can be used for future methods development and for revealing properties of epitope residues beyond the fact that epitopes are sites on the protein surface.

#### Scale-based methods: score value distributions

DiscoTope, PIER, ProMate and ConSurf assign to each protein residue a score reflecting the probability of that residue being a part of the protein interface or epitope. Details are provided in the Methods section. For the analysis of epitope residues versus non-epitope residues we used dataset #1, that is, representative antigen structures with epitopes mapped onto them. Here an epitope residue is an antigen residue known to be part of an epitope in any complex of this antigen with any antibody. Conversely a non-epitope residue implies an antigen residue which is not known to be part of a structural epitope. To simplify the calculation proteins with epitopes located on more than one protein chain were discarded from the analyses (there were 10 such proteins). As a result 52 protein antigens were analyzed [see Additional file 1].

**Figure 4:**
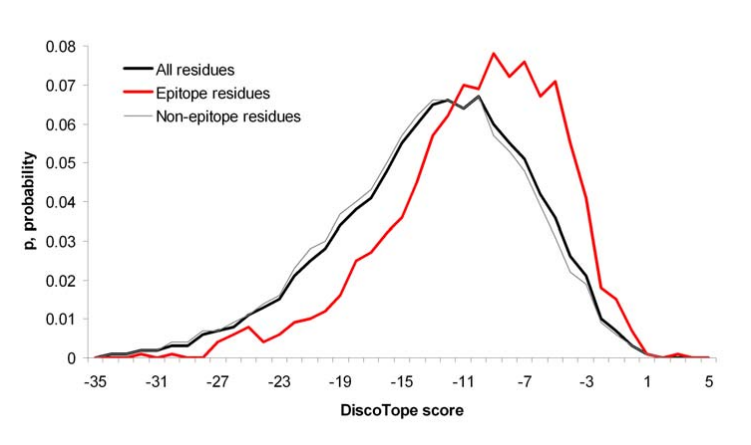
Distributions of DiscoTope scores for epitope, non-epitope and all protein residues.

**Figure 5:**
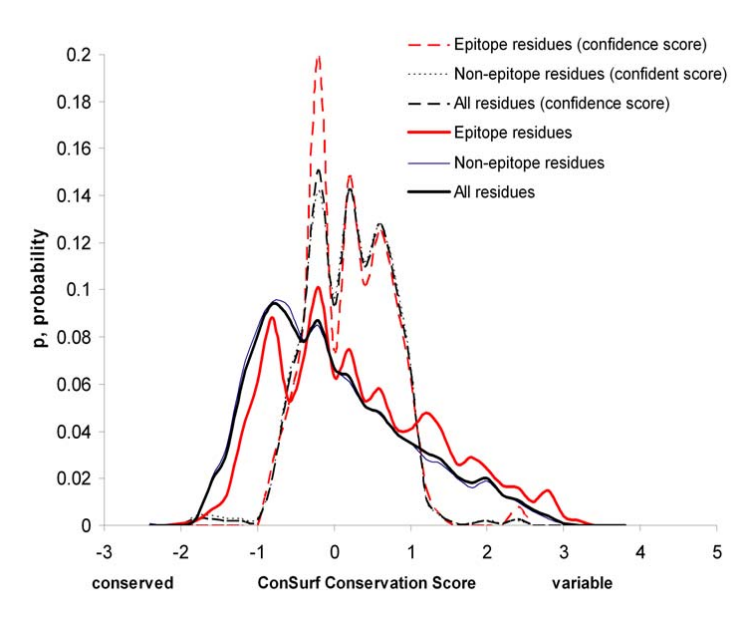
**Distribution of ConSurf scores for epitope and all protein residues**. For the definition of confidence score see the Methods section.

**Figure 6:**
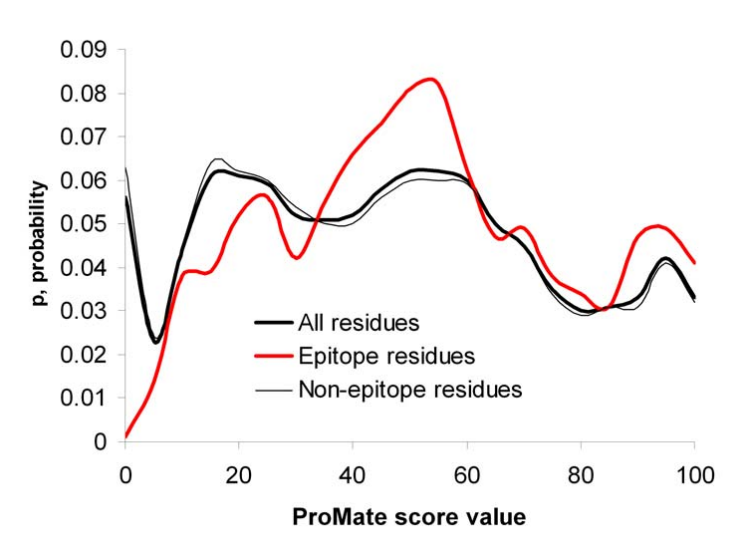
Distribution of ProMate scores for epitope, non-epitope and all protein residues.

**Figure 7:**
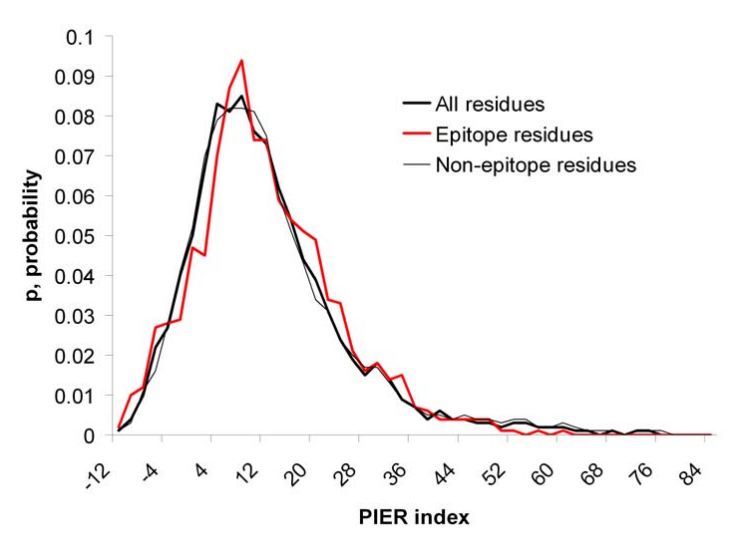
Distribution of PIER scores for epitope, non-epitope and all protein residues.

The score distributions for epitope, non-epitope and all protein residues were calculated for each method and are shown in Figures 4, 5, 6, 7. Distributions taking into account only surface residues were similar for all methods (results not shown). The definition of a surface residue is given in the Methods section.

DiscoTope, ProMate and ConSurf scores discriminate epitope versus non-epitope and versus all protein residues, while PIER and ConSurf confidence scores do not. Thus, as one can see in Figure 4, DiscoTope discriminates epitope residues (x¯
 MathType@MTEF@5@5@+=feaafiart1ev1aaatCvAUfKttLearuWrP9MDH5MBPbIqV92AaeXatLxBI9gBaebbnrfifHhDYfgasaacH8akY=wiFfYdH8Gipec8Eeeu0xXdbba9frFj0=OqFfea0dXdd9vqai=hGuQ8kuc9pgc9s8qqaq=dirpe0xb9q8qiLsFr0=vr0=vr0dc8meaabaqaciaacaGaaeqabaqabeGadaaakeaacuWG4baEgaqeaaaa@2E3D@ = -10.2, *s *= 5.4, number of residues N = 1,364) from non-epitope residues (x¯
 MathType@MTEF@5@5@+=feaafiart1ev1aaatCvAUfKttLearuWrP9MDH5MBPbIqV92AaeXatLxBI9gBaebbnrfifHhDYfgasaacH8akY=wiFfYdH8Gipec8Eeeu0xXdbba9frFj0=OqFfea0dXdd9vqai=hGuQ8kuc9pgc9s8qqaq=dirpe0xb9q8qiLsFr0=vr0=vr0dc8meaabaqaciaacaGaaeqabaqabeGadaaakeaacuWG4baEgaqeaaaa@2E3D@ = -13.3, *s *= 6.3, N = 9,713) (p < 0.001) and all antigen residues (x¯
 MathType@MTEF@5@5@+=feaafiart1ev1aaatCvAUfKttLearuWrP9MDH5MBPbIqV92AaeXatLxBI9gBaebbnrfifHhDYfgasaacH8akY=wiFfYdH8Gipec8Eeeu0xXdbba9frFj0=OqFfea0dXdd9vqai=hGuQ8kuc9pgc9s8qqaq=dirpe0xb9q8qiLsFr0=vr0=vr0dc8meaabaqaciaacaGaaeqabaqabeGadaaakeaacuWG4baEgaqeaaaa@2E3D@ = -13.0, *s *= 6.3, N = 11,077) (p < 0.001). These distributions are significantly different (p < 0.001) regardless of the epitope definition used. The ConSurf conservation score also discriminates epitope residues (x¯
 MathType@MTEF@5@5@+=feaafiart1ev1aaatCvAUfKttLearuWrP9MDH5MBPbIqV92AaeXatLxBI9gBaebbnrfifHhDYfgasaacH8akY=wiFfYdH8Gipec8Eeeu0xXdbba9frFj0=OqFfea0dXdd9vqai=hGuQ8kuc9pgc9s8qqaq=dirpe0xb9q8qiLsFr0=vr0=vr0dc8meaabaqaciaacaGaaeqabaqabeGadaaakeaacuWG4baEgaqeaaaa@2E3D@ = 0.273, *s *= 1.050, N = 1,119) versus non-epitope residues (x¯
 MathType@MTEF@5@5@+=feaafiart1ev1aaatCvAUfKttLearuWrP9MDH5MBPbIqV92AaeXatLxBI9gBaebbnrfifHhDYfgasaacH8akY=wiFfYdH8Gipec8Eeeu0xXdbba9frFj0=OqFfea0dXdd9vqai=hGuQ8kuc9pgc9s8qqaq=dirpe0xb9q8qiLsFr0=vr0=vr0dc8meaabaqaciaacaGaaeqabaqabeGadaaakeaacuWG4baEgaqeaaaa@2E3D@ = -0.049, *s *= 0.987, *p *< 0.001) and versus all antigen residues (x¯
 MathType@MTEF@5@5@+=feaafiart1ev1aaatCvAUfKttLearuWrP9MDH5MBPbIqV92AaeXatLxBI9gBaebbnrfifHhDYfgasaacH8akY=wiFfYdH8Gipec8Eeeu0xXdbba9frFj0=OqFfea0dXdd9vqai=hGuQ8kuc9pgc9s8qqaq=dirpe0xb9q8qiLsFr0=vr0=vr0dc8meaabaqaciaacaGaaeqabaqabeGadaaakeaacuWG4baEgaqeaaaa@2E3D@ = -0.007, *s *= 1.00, N = 8,684, *p *< 0.001) (Fig. 5). The same was true for epitope vs. all surface residues. Further, the confidence level did not change when the definition of surface residues and/or epitope residues was changed (data not shown). However, if only residues with ConSurf confidence score values were considered, no significant difference between epitope and other protein residues was observed (epitope residues: x¯
 MathType@MTEF@5@5@+=feaafiart1ev1aaatCvAUfKttLearuWrP9MDH5MBPbIqV92AaeXatLxBI9gBaebbnrfifHhDYfgasaacH8akY=wiFfYdH8Gipec8Eeeu0xXdbba9frFj0=OqFfea0dXdd9vqai=hGuQ8kuc9pgc9s8qqaq=dirpe0xb9q8qiLsFr0=vr0=vr0dc8meaabaqaciaacaGaaeqabaqabeGadaaakeaacuWG4baEgaqeaaaa@2E3D@ = 0.197, *s *= 0.539; non-epitope residues: x¯
 MathType@MTEF@5@5@+=feaafiart1ev1aaatCvAUfKttLearuWrP9MDH5MBPbIqV92AaeXatLxBI9gBaebbnrfifHhDYfgasaacH8akY=wiFfYdH8Gipec8Eeeu0xXdbba9frFj0=OqFfea0dXdd9vqai=hGuQ8kuc9pgc9s8qqaq=dirpe0xb9q8qiLsFr0=vr0=vr0dc8meaabaqaciaacaGaaeqabaqabeGadaaakeaacuWG4baEgaqeaaaa@2E3D@ = 0.194, *s *= 0.556, *p *> 0.05). For ProMate mean scores for epitope residues (x¯
 MathType@MTEF@5@5@+=feaafiart1ev1aaatCvAUfKttLearuWrP9MDH5MBPbIqV92AaeXatLxBI9gBaebbnrfifHhDYfgasaacH8akY=wiFfYdH8Gipec8Eeeu0xXdbba9frFj0=OqFfea0dXdd9vqai=hGuQ8kuc9pgc9s8qqaq=dirpe0xb9q8qiLsFr0=vr0=vr0dc8meaabaqaciaacaGaaeqabaqabeGadaaakeaacuWG4baEgaqeaaaa@2E3D@ = 52.8, *s *= 25.4, N = 1,363) were significantly higher than for all antigen residues (x¯
 MathType@MTEF@5@5@+=feaafiart1ev1aaatCvAUfKttLearuWrP9MDH5MBPbIqV92AaeXatLxBI9gBaebbnrfifHhDYfgasaacH8akY=wiFfYdH8Gipec8Eeeu0xXdbba9frFj0=OqFfea0dXdd9vqai=hGuQ8kuc9pgc9s8qqaq=dirpe0xb9q8qiLsFr0=vr0=vr0dc8meaabaqaciaacaGaaeqabaqabeGadaaakeaacuWG4baEgaqeaaaa@2E3D@ = 46.5, *s *= 28.1, N = 11,074) or non-epitope residues or all surface residues (*p *< 0.001) (Fig. 6). The PIER score does not discriminate epitope versus other antigen residues (epitope residues: x¯
 MathType@MTEF@5@5@+=feaafiart1ev1aaatCvAUfKttLearuWrP9MDH5MBPbIqV92AaeXatLxBI9gBaebbnrfifHhDYfgasaacH8akY=wiFfYdH8Gipec8Eeeu0xXdbba9frFj0=OqFfea0dXdd9vqai=hGuQ8kuc9pgc9s8qqaq=dirpe0xb9q8qiLsFr0=vr0=vr0dc8meaabaqaciaacaGaaeqabaqabeGadaaakeaacuWG4baEgaqeaaaa@2E3D@ = 11.9, *s *= 11.4, N = 1,363; non-epitope residues: x¯
 MathType@MTEF@5@5@+=feaafiart1ev1aaatCvAUfKttLearuWrP9MDH5MBPbIqV92AaeXatLxBI9gBaebbnrfifHhDYfgasaacH8akY=wiFfYdH8Gipec8Eeeu0xXdbba9frFj0=OqFfea0dXdd9vqai=hGuQ8kuc9pgc9s8qqaq=dirpe0xb9q8qiLsFr0=vr0=vr0dc8meaabaqaciaacaGaaeqabaqabeGadaaakeaacuWG4baEgaqeaaaa@2E3D@ = 12.6, *s *= 13.7; N = 8,221, *p *> 0.05) (Fig. 7).

These results suggest that epitope residues are less conservative according to the ConSurf evolutionary conservancy scores than protein surface residues in general at a 99.9% confidence level (*p *< 0.001). PIER, which is trained on 3D structures of all protein-protein complexes available in the PDB, could not distinguish epitopes from the rest of the protein surface. One possible explanation of this failure is that epitope residues do share some properties with residues having transient non-obligate hetero-interactions with other proteins. ProMate is trained using such complexes [55].

#### Criteria and dataset used in methods evaluation

There is no commonly acceptable standard for evaluating binding site prediction methods. Some authors measure performance on a per protein bases, measuring statistics across the dataset [49], while others measure performance on a per residue basis [54]. Some authors report sensitivity and specificity and measure the performance from the area under the ROC curve [49], while others consider only the sensitivity and positive predictive values and measure the method performance from the relative number of successful predictions in the test dataset [53].

Approaching the task of evaluation and comparison of different methods, we encountered a number of questions. How can we compare scale-based methods with patch prediction and docking methods? DiscoTope and ProMate predict one patch per protein, while other methods predict several patches, how can these be compared? Using a score value assigned by ProMate, DiscoTope, or ConSurf to a residue, all epitopes in the protein are taken into account, so can we say that the method predicts one epitope per protein? Is not the direct comparison of protein docking methods (ClusPro (DOT), PatchDock) versus patch-based prediction methods (DiscoTope, ProMate, CEP, PPI-PRED) questionable since the former methods are based on optimization of an interaction energy function, while the latter depend on training? Finally, docking methods require knowledge of the structures of both interacting proteins, antigen and antibody, while binding site prediction methods are based on the structure of the protein antigen alone and do not require knowledge of the antibody structure. Is this a fair comparison? Being aware of these questions and limitations, we applied various evaluation criteria in an attempt to provide a thorough and fair comparison of the methods.

The evaluation was performed on the dataset of representative epitopes, assuming any antigen residue which is not a part of a considered epitope is part of a non-epitope. We didn't discard non-epitope residues, which we know belong to some other epitope in the protein, because we assumed that a prediction program will predict an epitope in an antigen for which it doesn't have any additional information except its sequence and structure – this is how all evaluated methods were constructed. The analysis was performed using the representative epitopes from dataset #2 that were inferred from structures of one-chain (monomer) antigens in complexes with two-chain antibody fragments. There were 59 such epitopes in 48 antigens (Table 2).

**Table 2:** Results for representative epitope prediction by patch and protein docking methods

				**ProMate**		**PPI-PRED (1^st ^patch)**		**PPI-PRED (best patch)**		**PatchDock 1^st ^model**		**PatchDock best model of 10**			**ClusPro (DOT) 1^st ^model**		**ClusPro (DOT) best model of 10**			**CEP**			**DiscoTope (-7.7)**		
antigen	epitope	antigen size	epitope size	sensitivity	ppv	sensitivity	ppv	sensitivity	ppv	sensitivity	ppv	Model #	sensitivity	ppv	sensitivity	ppv	Model#	sensitivity	ppv	N predictions	sensitivity	ppv	Sensitivity	ppv	Is in training set?**&**
2ADF:A	2adf_A_HL	196	15	0	0	**0.8**	**0.67**	**0.8**	**0.67**	0	0	4	**0.4**	**0.29**	**0.67**	**0.5**	1	**0.67**	**0.5**	7	0.27	0.14	0.07	0.11	**-**
2ADF:A	1fe8_B_IM	196	20	0	0	**0.3**	**0.33**	**0.3**	**0.33**	0	0	2	**0.4**	**0.38**	**0.63**	**0.57**	1	**0.63**	**0.57**	7	**0.32**	**0.22**	0.15	0.33	*
1AFV:A	1afv_A_HL	151	14	0	0	0.57	0.15	0.57	0.15	**0.43**	**0.25**	1	**0.43**	**0.25**	0	0	1	0	0	6	**0.46**	**0.18**	0.43	0.1	**-**
1BGX:T	1bgx_T_HL	832	52	0	0	0.02	0.01	**0.33**	**0.11**	**0.79**	**0.77**	1	**0.79**	**0.77**	NA		NA			17	0.08	0.1	**0.37**	**0.16**	**-**
1E6J:P	1e6j_P_HL	210	12	0	0	0.08	0.03	**1**	**0.41**	0	0	9	**0.42**	**0.24**	0	0	7	**0.42**	**0.26**	5	0.33	0.08	0	0	**-**
1EGJ:A	1egj_A_HL	101	11	0.27	0.27	**0.64**	**0.44**	**0.64**	**0.44**	0.27	0.11	1	0.27	0.11	**0.73**	**0.8**	1	**0.73**	**0.8**	1	1	0.13	**0.91**	**0.16**	*
1FSK:A	1fsk_A_CB	159	17	0	0	0.59	0.17	0.59	0.17	**0.59**	**0.31**	1	**0.59**	**0.31**	0	0	8	**0.47**	**0.47**	6	0.12	0.11	**0.76**	**0.22**	*
1H0D:C	1h0d_C_BA	123	17	**0.65**	**0.85**	0.06	0.05	**0.59**	**1**	0	0	10	**0.53**	**0.5**	**0.59**	**0.56**	1	**0.59**	**0.56**	5	0.44	0.16	0.35	0.13	*
1I9R:A	1i9r_A_HL	146	18	0	0	0	0	0	0	0.17	0.14	3	**0.78**	**0.61**	0.11	0.14	5	**0.44**	**0.33**	7	0.11	0.1	0.17	0.23	**-**
1IQD:C	1iqd_C_BA	156	16	0.19	0.23	0	0	0	0	0.31	0.14	5	**0.94**	**0.83**	**0.38**	**0.32**	1	**0.38**	**0.32**	5	0.07	0.04	**0.56**	**0.3**	*
1JRH:I	1jrh_I_HL	108	15	0.07	0.1	**0.67**	**0.56**	**0.67**	**0.56**	**0.53**	**0.31**	1	**0.53**	**0.31**	**0.47**	**0.78**	1	**0.47**	**0.78**	1	0.73	0.15	**0.6**	**0.26**	**-**
1LK3:A	1lk3_A_HL	160	18	0	0	0	0	0	0	0.11	0.1	2	**0.67**	**0.57**	0.22	0.27	5	**0.56**	**0.62**	5	0.17	0.14	**0.61**	**0.32**	*
1MHP:B	1mhp_B_XY	192	19	0	0	0	0	**0.47**	**0.33**	**0.74**	**0.61**	1	**0.74**	**0.61**	**0.68**	**0.76**	1	**0.68**	**0.76**	2	0.11	0.13	**0.53**	**0.27**	**-**
1NL0:G	1nl0_G_HL	51	7	0	0	0	0	0	0	0.29	0.25	1	0.29	0.25	0.2	0.07	2	**1**	**0.5**	1	**0.71**	**0.42**	**0.57**	**0.33**	**-**
1NSN:S	1nsn_S_HL	149	18	0	0	0	0	**0.39**	**0.33**	**0.5**	**0.45**	1	**0.5**	**0.45**	0	0	4	**0.28**	**0.28**	3	0.06	0.03	0.39	0.14	**-**
1OAZ:A	1oaz_A_HL	123	17	**0.35**	**0.5**	**0.59**	**0.32**	**0.59**	**0.32**	**0.65**	**0.46**	1	**0.65**	**0.46**	**0.82**	**0.82**	1	**0.82**	**0.82**	5	**0.59**	**0.23**	0.29	0.2	*
1ORQ:C	1orq_C_BA	223	14	0	0	0	0	**0.5**	**0.14**	0	0	7	**0.5**	**0.26**	**0.29**	**0.33**	1	**0.29**	**0.33**	6	0.54	0.09	0	0	**-**
1ORS:C	1ors_C_BA	132	10	**0.6**	**0.46**	0	0	**0.7**	**0.3**	0.2	0.08	4	**0.6**	**0.24**	**0.4**	**0.24**	1	**0.4**	**0.24**	4	0.78	0.11	0	0	*
1PKQ:E	1pkq_E_BA	139	17	**0.35**	**0.5**	**0.3**	**0.31**	**0.3**	**0.31**	0.35	0.21	3	**0.65**	**0.55**	0.06	0.06	8	**0.29**	**0.29**	8	0.44	0.15	**0.47**	**0.24**	**-**
1RJL:C	1rjl_C_BA	95	13	0	0	0	0	0	0	0.31	0.19	6	**0.69**	**0.39**	0	0	1	0	0	5	0.58	0.14	0.54	0.23	**-**
1SY6:A	1sy6_A_HL	204	11	0	0	0	0	0	0	0	0	1	0	0	0	0	3	**0.45**	**0.24**	8	0.3	0.1	**0.91**	**0.14**	**-**
1TZI:V	1tzi_V_BA	102	4	0	0	0	0	**0.75**	**0.3**	0	0	1	0	0	0.5	0.09	6	**0.75**	**0.14**	3	1	0.05	0.5	0.05	**-**
1WEJ:F	1wej_F_HL	105	11	0	0	0	0	0	0	0.18	0.11	4	**0.73**	**0.44**	**0.45**	**0.36**	1	**0.45**	**0.36**	5	0.1	0.03	0.45	0.09	**-**
1YJD:C	1yjd_C_HL	140	14	0.14	0.17	**0.5**	**0.64**	**0.5**	**0.64**	**0.57**	**0.36**	1	**0.57**	**0.36**	0	0	4	**0.64**	**0.32**	6	0.36	0.11	0.21	0.16	**-**
1YNT:F	1ynt_F_BA	254	19	0	0	0	0	**0.79**	**0.58**	0	0	1	0	0	**0.74**	**0.88**	1	**0.74**	**0.88**	16	0.11	0.1	0	0	**-**
1YY9:A	1yy9_A_DC	624	20	0	0	0	0	0	0	0	0	1	0	0	NA		NA			22	0	0	0.2	0.07	**-**
1ZA3:R	1za3_R_HL	134	15	0.13	0.2	**0.47**	**0.41**	**0.47**	**0.41**	**0.73**	**0.39**	1	**0.73**	**0.39**	0	0	2	**1**	**0.88**	5	**0.57**	**0.2**	0.13	0.25	**-**
1ZTX:E	1ztx_E_HL	108	16	0.06	0.09	0	0	0	0	0.38	0.24	3	**0.44**	**0.37**	0	0	3	**0.56**	**0.45**	1	0.75	0.16	0.19	0.21	**-**
2JEL:P	2jel_P_HL	85	15	0	0	0	0	0	0	0	0	3	**0.4**	**0.38**	0	0	5	**0.47**	**0.37**	5	0.43	0.14	0.07	0.2	**-**
1A14:N	1a14_N_HL	388	17	0	0	0	0	**0.35**	**0.18**	0.18	0.12	6	**0.47**	**0.33**	0	0	1	0	0	11	0	0	**0.76**	**0.2**	*
1A14:N	1nca_N_HL	388	21	0	0	0	0	**0.52**	**0.33**	0	0	4	**1**	**0.81**	0	0	5	**0.86**	**0.86**	11	0	0	**0.62**	**0.2**	*
1RJC:B	1bvk_C_BA	129	17	0	0	0.12	0.09	**0.47**	**0.47**	0.06	0.05	1	0.06	0.05	0	0	3	**0.76**	**0.65**	3	0.24	0.1	0.29	0.23	*
1RJC:B	1jhl_A_HL	129	11	0	0	0.27	0.13	0.27	0.13	0	0	2	**0.82**	**0.36**	0	0	5	**0.45**	**0.33**	3	0.1	0.03	0.27	0.14	*
1RJC:B	1ndg_C_BA	129	21	**0.29**	**0.46**	**0.38**	**0.35**	**0.38**	**0.35**	**0.57**	**0.55**	1	**0.57**	**0.55**	0	0	2	**0.33**	**0.33**	3	0.43	0.23	**0.33**	**0.32**	*
1RJC:B	1p2c_C_BA	129	18	0.11	0.15	0.17	0.13	0.17	0.13	0.28	0.25	9	**0.33**	**0.33**	0.17	0.21	2	**0.67**	**0.6**	3	**0.56**	**0.26**	**0.5**	**0.41**	*
1JPS:T	1jps_T_HL	219	21	0.05	0.1	0.14	0.09	0.14	0.09	0.05	0.04	2	**0.57**	**0.32**	**0.86**	**0.9**	1	**0.86**	**0.9**	7	0.25	0.13	**0.33**	**0.19**	*
1AR1:B	1ar1_B_CD	298	16	0	0	0.06	0.03	0.06	0.03	0.06	0.04	1	0.06	0.04	0	0	1	0	0	12	0.13	0.05	0	0	*
1EO8:A	1eo8_A_HL	328	15	0	0	0	0	**0.87**	**0.23**	0	0	1	0	0	0	0	1	0	0	14	0.07	0.03	0	0	*
1EO8:A	1ken_A_HL	328	16	0	0	**0.69**	**0.23**	**0.69**	**0.23**	0	0	1	0	0	0	0	1	0	0	14	0.13	0.06	**0.56**	**0.13**	**-**
1EO8:A	1qfu_A_HL	328	19	0	0	0	0	**0.84**	**0.29**	0	0	7	**0.21**	**0.17**	0	0	3	**0.21**	**0.27**	14	0.11	0.05	0.11	0.03	*
1EO8:A	2vit_C_BA	328	18	0	0	**0.33**	**0.13**	**0.33**	**0.13**	0.22	0.02	2	**1**	**0.1**	0	0	10	**0.22**	**0.33**	14	0.18	0.08	0.17	0.04	**-**
1EZV:E	1ezv_E_XY	185	17	0	0	0	0	0	0	0.18	0.09	7	**0.53**	**0.53**	0	0	1	0	0	7	0.31	0.07	**1**	**0.29**	*
1OSP:O	1osp_O_HL	257	19	0	0	0.05	0.02	0.05	0.02	0	0	4	**0.63**	**0.31**	0	0	1	0	0	14	0.17	0.07	**0.53**	**0.17**	*
1OSP:O	1fj1_F_BA	257	17	0	0	0	0	**0.71**	**0.24**	0	0	5	**0.59**	**0.3**	**0.29**	**0.56**	1	**0.29**	**0.56**	14	0.25	0.09	**0.47**	**0.14**	*
1FNS:A	1fns_A_HL	196	12	0	0	0	0	0	0	0.17	0.07	6	**0.42**	**0.21**	0	0	8	**0.33**	**0.22**	7	0	0	**0.67**	**0.3**	*
1G9M:G	1g9m_G_HL	321	12	0	0	**0.67**	**0.12**	**0.67**	**0.12**	0.08	0.02	2	**0.33**	**0.1**	**0.5**	**0.29**	1	**0.5**	**0.29**	14	0.18	0.05	0.08	0.01	*
1G9M:G	2b4c_G_HL	321	17	0	0	**0.75**	**0.13**	**0.75**	**0.13**	**0.71**	**0.21**	1	**0.71**	**0.21**	**0.29**	**0.21**	1	**0.29**	**0.21**	14	0.09	0.03	0.08	0.01	**-**
1R3J:C	1r3j_C_BA	124	13	0	0	0	0	0	0	0.31	0.15	5	**0.85**	**0.85**	**0.85**	**0.92**	1	**0.85**	**0.92**	4	0.42	0.11	0.08	0.09	*
1N8Z:C	1n8z_C_BA	607	17	0.24	0.07	**0.3**	**0.38**	**0.3**	**0.38**	**0.24**	**0.09**	1	**0.24**	**0.09**	NA		NA			18	**0.18**	**0.1**	0.12	0.05	*
1N8Z:C	1s78_B_FE	607	23	0	0	0	0	0	0	0	0	1	0	0	NA		NA			18	0.05	0.03	**0.22**	**0.12**	**-**
1NFD:D	1nfd_D_HG	239	13	0	0	0	0	**0.92**	**0.32**	0.15	0.06	1	0.15	0.06	0	0	5	**0.31**	**0.18**	13	0.25	0.08	**0.77**	**0.16**	*
1TQB:A	1tqb_A_BC	102	18	0	0	0.28	0.29	**0.67**	**0.57**	**0.56**	**0.53**	1	**0.56**	**0.53**	0.17	0.2	3	**0.5**	**0.53**	2	0.11	0.08	0.78	0.21	*
1TXV:A	1txv_A_HL	452	19	0	0	0	0	0	0	0	0	1	0	0	0	0	7	**0.53**	**0.53**	18	0.11	0.06	**0.53**	**0.17**	*
1V7M:V	1v7m_V_HL	163	17	0	0	0	0	**0.35**	**0.32**	**0.41**	**0.39**	1	**0.41**	**0.39**	0.35	0.38	1	**0.35**	**0.38**	6	0.31	0.15	0.06	0.11	**-**
1XIW:A	1xiw_A_DC	105	18	0	0	0	0	0	0	**1**	**0.86**	1	**1**	**0.86**	0.83	0.79	1	**0.83**	**0.79**	26	0	0	**0.88**	**0.43**	**-**
1XIW:F	1xiw_F_DC	79	10	0.1	0.14	0	0	0	0	0.1	0.05	8	**0.6**	**0.32**	0.1	0.07	1	0.1	0.07	26	0	0	**0.4**	**0.44**	**-**
1Z3G:A	1z3g_A_HL	186	19	0.12	0.11	0.35	0.17	0.35	0.17	**0.53**	**0.3**	1	**0.53**	**0.3**	0	0	8	**0.26**	**0.25**	10	0.25	0.11	**0.35**	**0.35**	**-**
2AEP:A	2aep_A_HL	395	21	0	0	0	0	**0.48**	**0.3**	0	0	1	0	0	0.05	0.05	1	0.05	0.05	18	0.1	0.07	0.14	0.05	**-**
1R0A:B	1r0a_B_HL	429	11			**0.73**	**0.08**	**0.73**	**0.08**	**0.36**	**0.15**	1	**0.36**	**0.15**	0	0	1	0	0	10	0	0	**1**	**0.06**	*

'NA' means that results for the protein were not obtained.

Significant predictions (*p *≤ 0.05) are shown in bold.

& – Epitopes used in the DiscoTope training set are indicated by an asterisk; those not used in the training set are indicated by a hyphen.

The following parameters were used to evaluate the methods:

***Sensitivity (recall or true positive rate (TPR)) = TP/(TP + FN) ***– a proportion of correctly predicted epitope residues (TP) with respect to the total number of epitope residues (TP+FN).

***Specificity ****(or 1 – false positive rate (FPR)) ****= 1 - FP/(TN + FP) ***– a proportion of correctly predicted non-epitope residues (TN) with respect to the total number of non-epitope residues (TN+FP).

***Positive predictive value (PPV) (precision) = TP/(TP + FP) ***– a proportion of correctly predicted epitope residues (TP) with respect to the total number of predicted epitope residues (TP+FN).

***Accuracy (ACC) = (TP + TN)/(TP + FN + FP + TN) ***– a proportion of correctly predicted epitope and non-epitope residues with respect to all residues.

***Area under the ROC Curve (AUC) - ***A ROC curve is a graph representing a dependency of TPR versus FPR, or sensitivity versus specificity. The AUC measure is a widely used measure for immunoinformatics and bioinformatics methods; it has also been recommended for methods comparison in the recent report [25]. The AUC gives the general performance of the method; it is "equivalent to the probability that the classifier will rank a randomly chosen positive instance higher than a randomly chosen negative instance" [61].

***Success Rate - ***the number of epitopes from the dataset that were successfully predicted. While the AUC is a convenient and commonly used measure in immunoinformatics since many protein-protein binding site prediction methods, as well as three methods evaluated in the current work, ProMate, PPI-PRED, and CEP, used success rate as a measure of their performance, we considered it necessary to also calculate the methods success rates. While this measure is easily and naturally interpretable, it requires us to define the *successful prediction *and that can be done in many different ways. For this reason, many scientists try to avoid using this measure.

The statistical significance of a prediction, that is, the difference between observed and expected frequencies of an actual epitope/non-epitope residue in the predicted epitope/non-epitope, was determined by Fisher's exact test (right-tailed). The prediction was considered significant if the significance level was ≥95%, that is, the *P-value *was ≤ 0.05.

The above parameters were applied in evaluating the methods as follows:

(1) For the scale-based methods, ProMate, DiscoTope, ConSurf, and PIER, by varying the threshold values for score values classifying epitope residues from non-epitope residues, the AUC values have been calculated for each epitope.

(2) Success rates for all methods were calculated on a per protein bases taking into account one epitope per protein predicted with the highest significance. Such an approach assumes that if the epitope in a protein was successfully predicted, the prediction for the protein is successful. Criteria used for definition of successful prediction are discussed further.

(3) Patch prediction methods and protein-protein docking methods fall in the category of discrete classifiers, that is, they classify a residue as an epitope or non-epitope residue with no score assigned. Therefore, a ROC curve cannot be generated for these methods, only the AUC value can be estimated. Other statistics have also been obtained for these methods by averaging statistical values over epitopes and then calculating the overall statistical values over epitope and non-epitope residues in the dataset.

#### Prediction of individual epitopes

The results for each method in predicting 59 representative epitopes are given in Tables 2, 3 and supplementary materials [see Additional file 2]. For scale-based methods only the AUC values were computed (Table 3), while for patch prediction and docking methods all other statistics were produced (Table 2 and supplementary materials [see Additional file 2]).

**Table 3:** AUC values for representative epitopes

**antigen**	**epitope**	**PIER**	**ConSurf**	**ConSurf (confidence score)**	**ProMate (score)**	**ProMate (patch)**	**PPI-PRED (1^st ^patch)**	**PPI-PRED (best patch)**	**PatchDock 1^st ^model**	**PatchDock best model of 10**	**ClusPro (DOT) 1^st ^model**	**ClusPro (DOT) best model of 10**	**CEP**	**DiscoTope (score)**	**DiscoTope (-7.7)**	**Is in training set?&**
2ADF:A	2adf_A_HL	0.01	0.62	0.62	0.88	NA	0.88	0.88	0.45	0.66	0.81	0.81	0.57	0.53	0.51	**-**
2ADF:A	1fe8_B_IM	0.16	0.67	0.67	0.65	NA	0.62	0.62	0.45	0.67	0.79	0.79	0.60	0.81	0.56	*
1AFV:A	1afv_A_HL	0.78	0.48	0.41	0.60	0.49	0.62	0.62	0.65	0.65	0.43	0.43	0.63	0.53	0.52	**-**
1BGX:T	1bgx_T_HL	0.39	0.48	0.50	0.56	NA	0.42	0.58	0.89	0.89	NA	NA	0.52	0.74	0.62	**-**
1E6J:P	1e6j_P_HL	0.51	0.43	0.39	0.41	NA	0.46	0.96	0.43	0.67	0.47	0.67	0.55	0.23	0.30	**-**
1EGJ:A	1egj_A_HL	0.09	NA	NA	0.84	0.59	0.77	0.77	0.50	0.50	0.85	0.85	0.61	0.88	0.67	*
1FSK:A	1fsk_A_CB	0.85	0.31	0.33	0.54	0.44	0.62	0.62	0.72	0.72	0.44	0.70	0.50	0.82	0.71	*
1H0D:C	1h0d_C_BA	0.25	0.51	0.51	0.97	0.82	0.43	0.80	0.38	0.73	0.76	0.76	0.55	0.50	0.49	*
1I9R:A	1i9r_A_HL	0.47	0.72	0.74	0.43	0.45	0.45	0.45	0.51	0.86	0.51	0.66	0.48	0.71	0.55	**-**
1IQD:C	1iqd_C_BA	0.10	0.80	0.81	0.74	0.56	0.44	0.44	0.55	0.96	0.64	0.64	0.45	0.78	0.71	*
1JRH:I	1jrh_I_HL	0.57	0.62	NA	0.49	0.49	0.79	0.79	0.67	0.67	0.72	0.72	0.53	0.62	0.67	**-**
1LK3:A	1lk3_A_HL	0.71	0.72	0.76	0.38	0.45	0.40	0.40	0.49	0.81	0.57	0.76	0.52	0.81	0.72	*
1MHP:B	1mhp_B_XY	0.15	0.44	0.42	0.88	NA	0.42	0.68	0.85	0.85	0.83	0.83	0.52	0.81	0.69	**-**
1NL0:G	1nl0_G_HL	0.23	NA	NA	0.16	0.45	0.42	0.42	0.58	0.58	0.46	0.95	0.78	0.61	0.69	**-**
1NSN:S	1nsn_S_HL	0.76	0.75	0.78	0.26	0.45	0.40	0.64	0.71	0.71	0.44	0.59	0.41	0.58	0.53	**-**
1OAZ:A	1oaz_A_HL	0.17	0.33	0.25	0.85	0.65	0.70	0.70	0.77	0.77	0.90	0.90	0.64	0.61	0.55	*
1ORQ:C	1orq_C_BA	0.65	0.60	0.61	0.55	0.44	0.33	0.64	0.42	0.70	0.63	0.63	0.60	0.48	0.44	**-**
1ORS:C	1ors_C_BA	0.59	NA	NA	0.96	0.77	0.39	0.78	0.51	0.72	0.65	0.65	0.66	0.50	0.42	*
1PKQ:E	1pkq_E_BA	0.48	0.69	0.70	0.76	0.65	0.60	0.60	0.59	0.79	0.46	0.60	0.56	0.71	0.63	**-**
1RJL:C	1rjl_C_BA	0.64	0.51	0.48	0.33	0.48	0.37	0.37	0.55	0.76	0.39	0.39	0.53	0.73	0.62	**-**
1SY6:A	1sy6_A_HL	0.83	0.45	NA	0.54	0.49	0.43	0.43	0.43	0.43	0.45	0.68	0.58	0.82	0.80	**-**
1TZI:V	1tzi_V_BA	0.20	0.49	0.55	0.56	0.45	0.37	0.84	0.36	0.36	0.64	0.78	0.60	0.52	0.54	**-**
1WEJ:F	1wej_F_HL	0.67	0.82	0.83	0.33	0.44	0.45	0.45	0.50	0.81	0.68	0.68	0.39	0.47	0.45	**-**
1YJD:C	1yjd_C_HL	0.44	0.53	0.54	0.80	0.53	0.73	0.73	0.73	0.73	0.44	0.74	0.52	0.58	0.54	**-**
1YNT:F	1ynt_F_BA	0.31	NA	NA	0.69	NA	0.44	0.87	0.44	0.44	0.87	0.87	0.51	0.49	0.46	**-**
1YY9:A	1yy9_A_DC	0.46	0.74	0.75	0.20	0.45	0.46	0.46	0.47	0.47	NA	NA	0.48	0.68	0.55	**-**
1ZA3:R	1za3_R_HL	0.29	0.72	0.77	0.61	0.53	0.69	0.69	0.80	0.80	0.45	0.99	0.65	0.69	0.54	**-**
1ZTX:E	1ztx_E_HL	0.73	0.63	0.63	0.37	0.48	0.43	0.43	0.59	0.66	0.39	0.72	0.54	0.47	0.54	**-**
2JEL:P	2jel_P_HL	0.58	0.70	0.70	0.59	NA	0.41	0.41	0.36	0.63	0.40	0.65	0.44	0.66	0.51	**-**
1A14:N	1a14_N_HL	0.69	0.75	0.76	0.38	0.45	0.46	0.64	0.56	0.72	0.47	0.47	0.47	0.87	0.81	*
1A14:N	1nca_N_HL	0.56	0.67	0.69	0.30	0.45	0.46	0.73	0.46	1.00	0.47	0.93	0.47	0.84	0.74	*
1RJC:B	1bvk_C_BA	0.61	0.62	0.61	0.66	0.44	0.47	0.69	0.44	0.44	0.44	0.85	0.46	0.66	0.57	*
1RJC:B	1jhl_A_HL	0.45	0.73	0.73	0.51	0.44	0.55	0.55	0.42	0.84	0.44	0.68	0.39	0.79	0.55	*
1RJC:B	1ndg_C_BA	0.46	0.66	0.65	0.66	0.61	0.62	0.62	0.74	0.74	0.40	0.60	0.57	0.74	0.60	*
1RJC:B	1p2c_C_BA	0.70	0.55	0.57	0.48	0.51	0.49	0.49	0.57	0.61	0.54	0.80	0.65	0.74	0.69	*
1JPS:T	1jps_T_HL	0.49	0.63	0.72	0.77	0.50	0.49	0.49	0.46	0.72	0.92	0.92	0.54	0.62	0.59	*
1AR1:B	1ar1_B_CD	0.87	0.62	0.64	0.15	0.46	0.48	0.48	0.49	0.49	0.47	0.47	0.49	0.57	0.45	*
1EO8:A	1eo8_A_HL	0.43	0.64	0.65	0.67	0.49	0.42	0.87	0.45	0.45	0.48	0.48	0.48	0.27	0.39	*
1EO8:A	1ken_A_HL	0.32	0.61	0.62	0.59	0.49	0.79	0.79	0.44	0.44	0.49	0.49	0.51	0.76	0.68	**-**
1EO8:A	1qfu_A_HL	0.54	0.64	0.64	0.60	0.49	0.42	0.86	0.45	0.58	0.48	0.59	0.50	0.38	0.44	*
1EO8:A	2vit_C_BA	0.51	0.56	0.63	0.48	0.49	0.60	0.60	0.29	0.68	0.48	0.59	0.54	0.58	0.48	**-**
1EZV:E	1ezv_E_XY	0.84	0.62	0.64	0.23	0.44	0.42	0.42	0.50	0.74	0.48	0.48	0.47	0.85	0.88	*
1OSP:O	1osp_O_HL	0.90	0.41	0.38	0.82	NA	0.40	0.40	0.46	0.76	0.48	0.48	0.49	0.76	0.66	*
1OSP:O	1fj1_F_BA	0.17	0.50	0.50	0.62	NA	0.37	0.77	0.45	0.75	0.64	0.64	0.54	0.68	0.63	*
1FNS:A	1fns_A_HL	0.50	0.57	0.57	0.40	NA	0.45	0.45	0.52	0.66	0.45	0.63	0.44	0.92	0.78	*
1G9M:G	1g9m_G_HL	0.17	0.49	0.48	0.68	0.45	0.74	0.74	0.47	0.61	0.73	0.73	0.53	0.44	0.43	*
1G9M:G	2b4c_G_HL	0.13	0.46	0.44	0.68	0.45	0.78	0.78	0.79	0.79	0.62	0.62	0.48	0.43	0.43	**-**
1R3J:C	1r3j_C_BA	0.84	0.81	0.81	0.53	0.45	0.42	0.42	0.56	0.92	0.92	0.92	0.52	0.72	0.49	*
1N8Z:C	1n8z_C_BA	0.30	0.46	0.46	0.82	0.57	0.64	0.64	0.59	0.59	NA	NA	0.57	0.59	0.53	*
1N8Z:C	1s78_B_FE	0.16	0.56	0.57	0.60	0.45	0.49	0.49	0.47	0.47	NA	NA	0.50	0.55	0.58	**-**
1NFD:D	1nfd_D_HG	0.90	0.73	0.71	0.34	0.46	0.43	0.90	0.51	0.51	0.46	0.62	0.55	0.88	0.77	*
1TQB:A	1tqb_A_BC	0.44	0.42	0.44	0.33	0.43	0.57	0.78	0.73	0.73	0.51	0.70	0.41	0.59	0.57	*
1TXV:A	1txv_A_HL	0.64	0.88	0.89	0.59	0.45	0.43	0.43	0.47	0.47	0.47	0.75	0.52	0.87	0.71	*
1V7M:V	1v7m_V_HL	0.67	0.59	NA	0.37	0.48	0.45	0.63	0.67	0.67	0.64	0.64	0.56	0.47	0.50	**-**
1XIW:A	1xiw_A_DC	0.85	0.76	0.90	0.31	0.44	0.41	0.41	0.99	0.99	0.89	0.89	0.45	0.87	0.83	**-**
1XIW:F	1xiw_F_DC	0.69	0.65	0.74	0.60	0.51	0.41	0.41	0.42	0.71	0.45	0.45	0.48	0.59	0.66	**-**
1Z3G:A	1z3g_A_HL	0.48	0.65	0.59	0.34	0.51	0.59	0.59	0.70	0.70	0.43	0.59	0.53	0.66	0.64	**-**
2AEP:A	2aep_A_HL	0.68	0.50	0.51	0.43	0.45	0.46	0.71	0.46	0.46	0.50	0.50	0.52	0.70	0.49	**-**
1R0A:B	1r0a_B_HL	0.12	0.51	0.54	0.02	NA	0.76	0.76	0.65	0.65	0.47	0.47	0.45	0.94	0.79	*

'NA' means that results for the epitope/protein were not obtained.

& – Epitopes used in the DiscoTope training set are indicated by an asterisk; those not used in the training set are indicated by a hyphen.

DiscoTope and ProMate predict only one epitope per protein. ClusPro and PatchDock rank predicted models starting from the model with the best score. For these methods, the first (by rank) prediction was considered. If it was not significant (*p *> 0.05), the next by rank significant prediction (not exceeding the 10 best predictions) was reported in Table 2. Since the number of epitopes predicted by CEP in a protein varies (Table 2) and they are not ranked, the average prediction was reported for each epitope. More detailed statistics on the prediction results is provided in the supplementary table  [see Additional file 2].

No one epitope was predicted by all methods (Table 2). Some epitopes, for example, HyHEL-8 on HEL [PDB:1NDG] and 8–18C5 on myelin oligodendrocyte glycoprotein [PDB:1PKQ], were predicted by all methods except CEP (Table 2). Two epitopes, cetuximab on EGFR [PDB:1YY9] and 7E2C50S on cytochrome c oxidase [PDB:1AR1], appeared to be difficult to predict; they could probably be predicted using the ConSurf average score in combination with a patch generation method. The extracellular region of EGFR [PDB:1YY9] is a large (624 aa) loosely-packed multi-domain protein with a lot of loops and hence epitope recognition appears difficult. Similarly, recognition of epitopes on subunit II of cytochrome c oxidase [PDB:1AR1] appears problematic because the protein possesses long protruded α-helixes.

The lower specificity of CEP and DiscoTope [see Additional file 2] results from these methods predicting larger epitopes (average size of predicted epitope by CEP is 40 residues, DiscoTope (-7.7) – 43 and DiscoTope (-10.5) – 80 residues) in comparison with other methods. The average size of predicted epitope size for PatchDock is 29 residues, ClusPro (DOT) is 17 residues, and PPI-PRED is 32 residues. The size of actual epitopes in the dataset varies from 4 to 52 residues (x¯
 MathType@MTEF@5@5@+=feaafiart1ev1aaatCvAUfKttLearuWrP9MDH5MBPbIqV92AaeXatLxBI9gBaebbnrfifHhDYfgasaacH8akY=wiFfYdH8Gipec8Eeeu0xXdbba9frFj0=OqFfea0dXdd9vqai=hGuQ8kuc9pgc9s8qqaq=dirpe0xb9q8qiLsFr0=vr0=vr0dc8meaabaqaciaacaGaaeqabaqabeGadaaakeaaieWacuWF4baEgaqeaaaa@2E45@ = 16, *s *= 6). However, it should be emphasized that if the most of the methods considered were designed to predict an epitope as a whole single entity, DiscoTope focuses on the prediction of individual epitope residues that can be part of several different epitopes in the same protein. Therefore, the average size of the epitope predicted by DiscoTope is large; moreover, the predicted epitope residues can be located too far from each on the protein surface to form a single epitope.

#### Overall performance of each method

The overall performance of each method have been compared first using average AUC values for all methods and then calculating all other statistics for patch prediction and protein-protein docking methods. Both comparisons were made on different subsets of representative epitopes from dataset #2.

Calculating AUC values for all methods, we discarded from the analysis the proteins for which any method didn't produced a result (ConSurf, ProMate, and ClusPro (DOT) were not able to predict epitopes for several proteins, see Methods). The final subset contained 42 epitopes from Table 2 of which 21 epitopes were not used for DiscoTope training. All other methods didn't use any epitopes for training.

**Figure 8:**
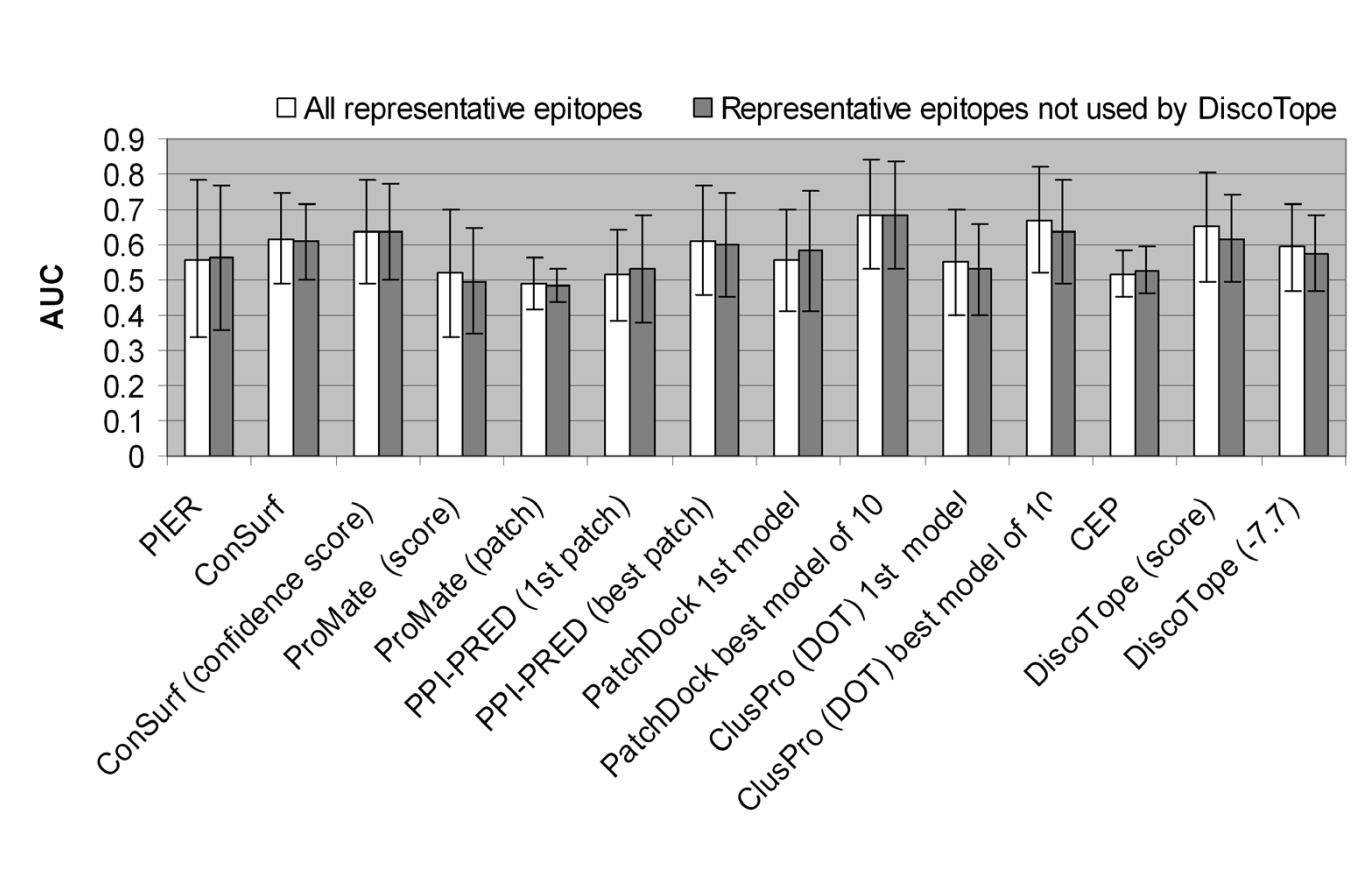
**Average AUC values for each method**. Vertical bars show one standard deviation.

AUC values averaged on subsets of 42 and 21 epitopes are shown in Figure 8. ConSurf, DiscoTope, PPI-PRED and docking methods, when the 10 best models were considered, demonstrated average AUC values above 0.6, that is, poor or mediocre performance. PatchDock was the best, giving an AUC of 0.69. All other methods performed close to random (Fig. 8). DiscoTope gave AUC values of 0.65 and 0.62 on all representative epitopes and those that were not used by the method for training, respectively. When DiscoTope performance was evaluated by the authors of the method [49], it gave an AUC value of 0.71 averaged over the five evaluation sets used for cross-validation.

For patch prediction and docking methods, to avoid the problematic comparison of methods predicting one epitope per protein with those that predict several epitopes, all epitopes from proteins with more than one epitope have been removed from dataset #2. Epitopes from proteins for which any method did not produce the prediction have also been discarded. The following statistics were calculated on the resulting subset of epitopes.

**Figure 9:**
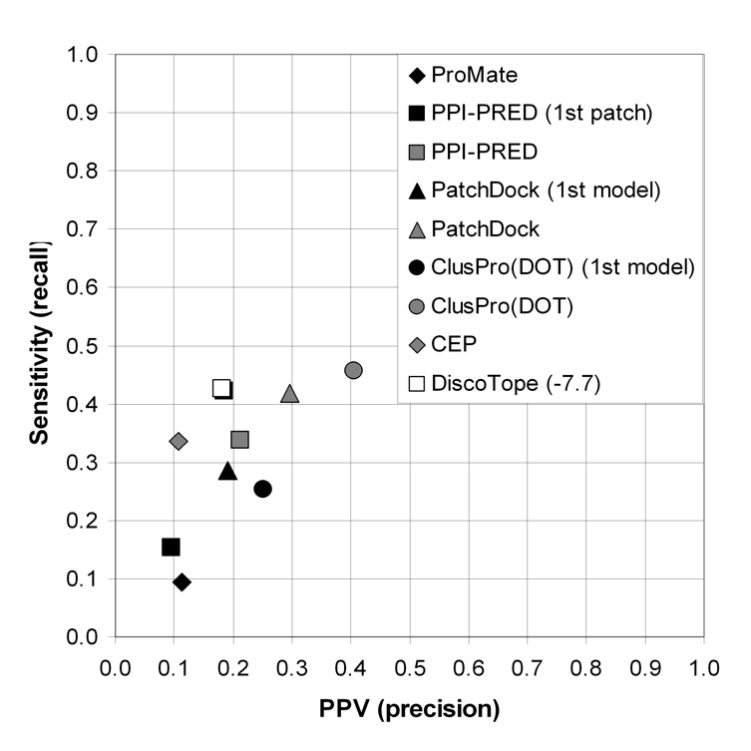
Overall methods performance measured as average sensitivity and PPV values.

First, FP, FN, TP, and TN values were summarized for the whole pool of epitopes, and sensitivity, specificity, accuracy, PPV, and AUC values calculated for each method (Table 4, upper part). AUC values obtained in this way were close to those demonstrated in Fig. 9. The best performers were docking methods PatchDock and DOT when the top ten models were considered, giving AUC values of 0.66 and 0.69, respectively (Table 4). Among the methods producing one prediction per protein, DiscoTope was rated the best by with an AUC of 0.60.

**Table 4:** Overall performance of patch prediction and protein-protein docking methods

**statistics**	**ProMate**	**PPI-PRED 1^st ^patch**	**PPI-PRED best patch**	**PatchDock 1^st ^model**	**PatchDock best model of 10**	**ClusPro (DOT) 1^st ^model**	**ClusPro (DOT) best model of 10**	**CEP**	**DiscoTope (-7.7)**
**sensitivity**	0.091	0.153	0.331	0.300	0.425	0.258	0.453	0.310	0.416
**1-specificity**	0.083	0.161	0.135	0.135	0.114	0.079	0.067	0.223	0.214
**PPV**	0.101	0.083	0.188	0.175	0.262	0.235	0.390	0.110	0.155
**accuracy**	0.841	0.780	0.819	0.816	0.846	0.863	0.892	0.739	0.754
**AUC**	0.504	0.496	0.598	0.583	0.656	0.589	0.693	0.544	0.601

**P-value**	0.27	1.0	7.8E-30	9.0E-23	<1.0E-50	7.9E-34	<1.0E-50	4.3E-06	4.1E-25

**Statistics averaged over epitopes**									
**sensitivity**	0.09 ± 0.17	0.15 ± 0.24	0.34 ± 0.32	0.27 ± 0.24	0.42 ± 0.29	0.25 ± 0.31	0.46 ± 0.28	0.34 ± 0.28	0.43 ± 0.31
**1-specificity**	0.08 ± 0.03	0.16 ± 0.07	0.14 ± 0.07	0.15 ± 0.06	0.13 ± 0.07	0.10 ± 0.07	0.08 ± 0.05	0.28 ± 0.20	0.22 ± 0.15
**PPV**	0.11 ± 0.20	0.10 ± 0.17	0.21 ± 0.24	0.18 ± 0.19	0.30 ± 0.25	0.25 ± 0.33	0.41 ± 0.29	0.11 ± 0.08	0.18 ± 0.12
**accuracy**	0.83 ± 0.05	0.77 ± 0.07	0.81 ± 0.08	0.80 ± 0.08	0.83 ± 0.09	0.84 ± 0.09	0.88 ± 0.07	0.69 ± 0.17	0.74 ± 0.12
**AUC**	0.51 ± 0.09	0.50 ± 0.13	0.60 ± 0.17	0.56 ± 0.11	0.64 ± 0.17	0.58 ± 0.17	0.69 ± 0.15	0.53 ± 0.08	0.60 ± 0.13

Second, statistics were averaged over epitopes (Table 4, lower part). The overall performance was poor for all methods. The best performance demonstrated by docking methods (when the 10 best models were considered) was 41% PPV (precision) and 46% sensitivity (recall) for ClusPro(DOT) and 30% PPV and 42% sensitivity for PatchDock. Among the methods producing one prediction per protein, DiscoTope was rated the best by sensitivity (43% sensitivity at 18% PPV) and ClusPro(DOT) first model by PPV (25% sensitivity and 25% PPV) (Fig. 9).

#### Comparison of success rates

**Figure 10:**
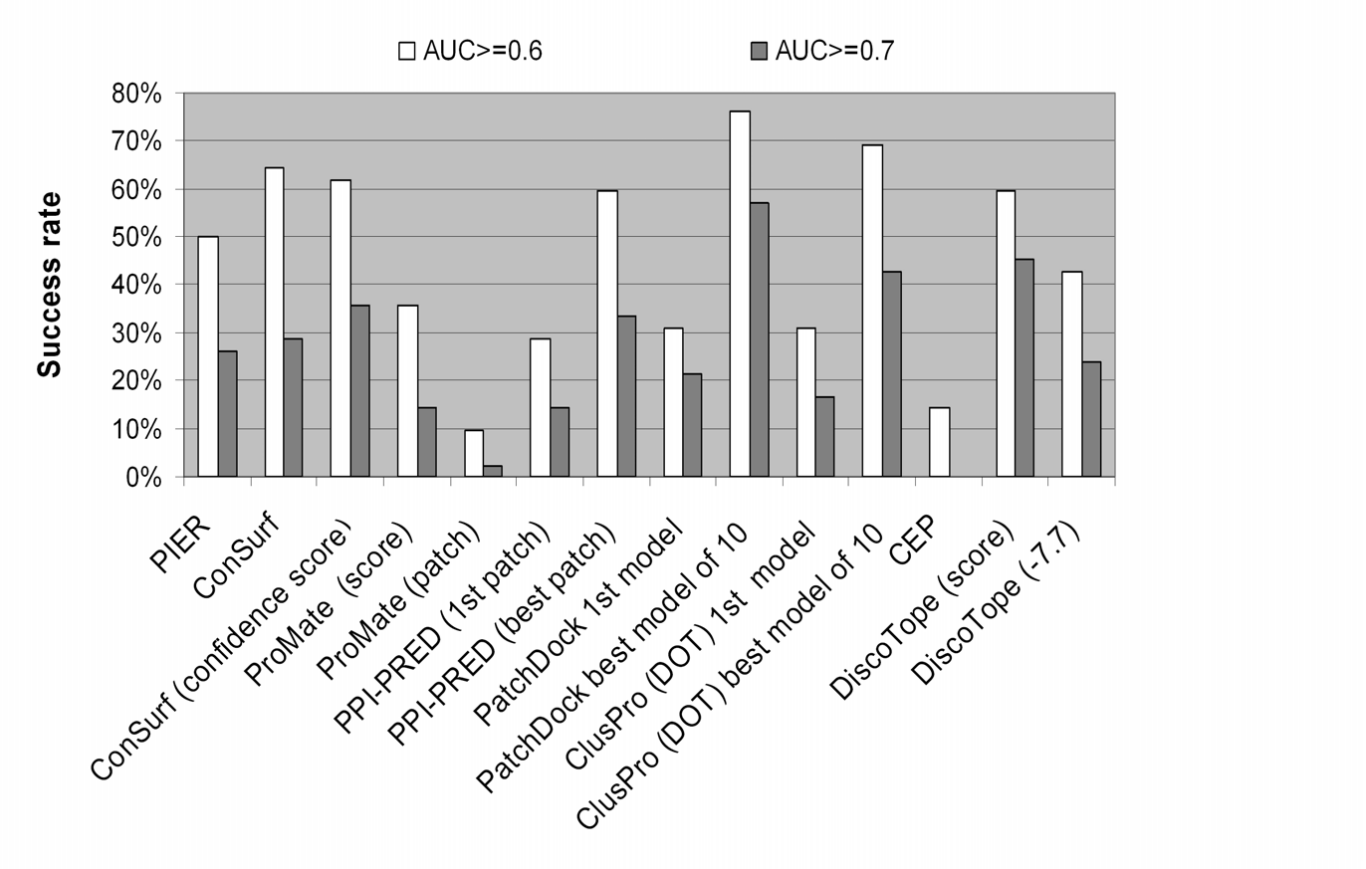
Proportion of successfully predicted epitopes.

Since patch prediction methods used in the current analysis used success rate as a performance measure, we additionally calculated the methods success rate on the subset of 42 epitopes used for overall methods comparison above. The prediction of each epitope was deemed successful if the AUC value was above a threshold value of 0.6 or 0.7. The results are presented in Fig. 10.

The proportion of epitopes predicted with an AUC ≥ 0.6 for the scale-based methods (PIER, ConSurf, ProMate, and DiscoTope) and the methods providing only one prediction per protein (ProMate, DiscoTope, PPI-PRED first prediction, PatchDock first model and ClusPro(DOT) first model) was lower than 50% except for ConSurf and DiscoTope methods, which showed success rates of approximately 60% (white bars in Fig. 10). Among the methods predicting several epitopes per protein (PPI-PRED, PatchDock, ClusPro(DOT), and CEP) PatchDock performed best with >75% successful predictions at an AUC ≥ 0.6 and 55% at an AUC ≥ 0.7 (Fig. 10).

PPI-PRED predicted 75% of protein-protein binding interfaces successfully, with a specificity over 50% and sensitivity over 20%, values previously used to claim success [53]. The ProMate's authors reported a success rate for protein-protein binding site prediction of 70% [55], while application of the criteria used in PPI-PRED gave ProMate's success rate as 36% [53]. According to our data [see Additional file 2], the prediction with an AUC ≥ 0.6 corresponded to a significant prediction (P-value < 0.05) at a sensitivity >30%. Using an AUC ≥ 0.6 as a criterion of successful prediction, PPI-PRED gave 60% and ProMate 35% successful predictions, respectively (Fig. 10). Neither ProMate nor PPI-PRED used antibody-protein interfaces for their methods development; nevertheless, they predicted epitopes with a success rate comparable to those for prediction of protein interfaces.

Epitopes and other protein-protein interfaces indeed share many properties. Thus, Blythe [62] compared 57 protein-protein binding interfaces of 44 proteins from the dataset used for ProMate development [55] with epitopes and paratopes inferred from X-ray structures of 37 complexes calculating the following interface properties: amino acid composition, hydrophobicity by the Eisenberg's scale [63], amino acid contribution to form inter-molecular hydrogen bonds, residue evolutionary conservancy, and several geometrical parameters, such as planarity and complementarity of interfaces. Epitopes and non-obligate heterodimer interfaces were very similar considering all the aforementioned properties except residue conservancy; epitope residues were more variable than heterodimer interfaces [62]. The current work additionally demonstrates that, on average, epitope residues are significantly less conservative than protein surface residues. Indeed, protein-protein interaction sites are under evolutionary pressure to be more conserved than protein surface residues on average. While antibody-antigen interactions are not under evolutionary pressure, they are under the selection pressure from the host immune system. This selection pressure is assumed to cause polymorphisms in pathogens and to explain the variability of immune epitopes.

## Conclusion

Benchmark datasets for use in B cell structural epitope prediction have been constructed and made available. Using these benchmark data, eight publicly available web servers and their associated methods were evaluated. Several schemes for methods evaluation were considered.

The overall performance was poor for all methods and did not exceed an average AUC of 0.7 and 40% positive predictive value (precision) at 46% sensitivity (recall). The values of the area under the receiver operating characteristic (ROC) curve for the evaluated methods were about 0.6 for ConSurf, DiscoTope, and PPI-PRED (when all predictions were considered) and above 0.65 but not exceeding 0.7 for protein-protein docking methods when the best of the top ten models for the bound docking were considered. Certainly a best case, since under real conditions many more models would be presented. Other methods, PIER, ProMate (both scores and patch prediction), CEP, PPI-PRED first patch, and the first models of docking methods, performed close to random. Despite the fact that structural epitopes and protein-protein non-obligate transient heterodimer interfaces share many properties, protein-protein binding site prediction methods were poor epitope predictors.

When the top ten models and bound docking were considered, the docking methods performed the best, especially PatchDock, where success can be explained by application of the CDR filter, which the DOT algorithm does not use. Independent evaluation of PPI-PRED and four docking algorithms (DOT, PatchDock, ZDOCK, and webGRAMM) made by Martin Blythe [62] and not available to us until the peer-reviewing stage of the manuscript agrees with the results presenting in the current work. Using the Matthew's Correlation Coefficient (MCC), Blythe measured the correlation between predicted and structural epitopes and paratopes inferred from 37 antibody-protein complexes. For the first models, all evaluated methods demonstrated near random correlations. Likewise, when the top ten models for each complex were considered, low and negative MCC values prevailed over positive values for all algorithms except PatchDock. Further experiments demonstrated that using the CDR filter may improve the prediction. Thus, using predefined CDRs for antibodies, the DOT method significantly improved and showed MCC values comparable with those for PatchDock [62].

Obviously, unbound docking would have more practical value for epitope prediction than bound docking. However, the performance of unbound docking for antibody-antigen interactions, as was shown by the authors of PatchDock, was unsatisfactory in comparison to bound docking and other protein-protein interaction methods [64]. While the bound docking considered in this work has no practical value for epitope prediction, it needs to be benchmarked to further improve unbound docking algorithms and tune them for modeling antibody-antigen complexes.

Currently the problem of B-cell epitope prediction is far from solved: structure-based method for prediction of discontinuous epitopes perform on the same level as sequence-based methods for prediction of continuous epitopes giving the area under the receiver operating characteristic curve (AUC) values of approximately 0.60 [25].

Three definitions of an epitope inferred from the X-ray structure of antibody-protein complexes were considered, but this made no significant difference to the predictions. Hence, we finally considered an epitope residue as the protein antigen residue for which any atom is separated from any antibody atom by a distance ≤ 4Å.

Currently, each method requires writing a separate parser taking into account different representations of the output data. There is a need to develop a common format for output data generated by both scale-based and patch generation tools that is easily interpreted by both a human and computer.

Given these shortcomings and current success rates, how can epitope prediction be improved? The availability of larger datasets containing only well-defined epitopes inferred from X-ray structures of antibody-protein complexes, which are then used for training, would help. This will come over time as the PDB continues to grow at a rapid rate. This need, in the context of continuous epitope prediction, has been noted by others [25]. The performance of docking algorithms might be improved by tuning them specifically for antibody-antigen complexes. Existing B-cell epitope prediction methods utilize only a few features characterizing epitopes (amino acid propensities, residue solvent accessibility, spatial distribution, and inter-molecular contacts). Therefore, another possible way for improving the prediction would be to utilize more features that discriminate epitopes from non-epitopes, for example, the evolutionary conservation score. This assumes that an epitope is indeed a discreet entity based upon what we know about proteins today. Perhaps the more fundamental question is whether it makes sense to consider a B-cell epitope a discrete feature of a protein at all? Time will tell as more X-ray structures on antibody-protein complexes become available.

## Methods

### Surface residue

is defined as a protein residue with a relative ASA of ≥ 1% as calculated by the program NACCESS. This cut-off was previously used by Jones & Thornton [46].

### Data sets compilation

169 structures of protein antigens (length >30 amino acids) in complex with antibody fragments have been manually collected from the PDB [41] of January 2006 at a resolution ≤4Å. Every structure has been manually curated within the IEDB database [1] and inspected using the EpitopeViewer visualization tool developed by the authors [65]. Structures in which the antibody binds antigen but involves no CDR residues have been excluded from the analysis; there were four such structures [PDB: 1MHH, 1HEZ, 1DEE, 1IGC]. If a structure contained several complexes in one asymmetric unit (there were 46 such structures in 165) and the authors of the structure observed no structural difference between these complexes, only one complex was selected – those that were specified as a reference complex by the authors of the article describing the structure (primary citation in the PDB); there were 18 such structures out of 46. If the authors didn't provide this information, all complexes in the structure were considered for analysis. The authors of a few structures clearly stated in their papers that antibody-protein contacts in the complexes were different: [PDB: 1MLC, 1NFD, 1OB1, 1P2C, 1QFW]. This initial curation has performed in order to correctly assign the protein-antibody complexes and decrease the number of individual complexes analyzed from 226 to 187 from a total of 169 structures. A total of 24 complexes were formed by one-chain antibody fragments and 163 complexes by two-chain antibody fragments. Alignment of protein chains was performed using the CE algorithm [58].

### Web-servers evaluation

The publicly available web-servers implementing 3D structure-based methods for protein-protein binding site and/or discontinuous epitope prediction were identified through PubMed and web searches. Eight web-servers were selected for evaluation (Table 1). The servers were tested between June and September of 2006, and results reflect the method implemented by the servers at that time. In all cases the default parameters provided by each server were used.

PPI-PRED provides up to three surface patches predicted as putative binding sites. The batch mode for data submission was used.

CEP provides residues forming the putative conformational epitopes (there could be more than 20 predictions per protein antigen). CEP includes residues with accessibility less than 25%. In this work, only residues with accessibility more than 25% were considered as a part of the epitope.

DiscoTope assigns a score to each protein residue that reflects the probability of that residue being part of an epitope and also provides a list of residues included in the predicted epitope (patch). DiscoTope predicts one epitope per protein.

ProMate returns results in four different formats. In this work, the two formats provided for each residue patch/non-patch identifier and residue interface probability were used. The batch version of ProMate, MultiProMate, was used.

PIER returns a list of residues with assigned PIER index values indicating how likely a particular residue is to be involved in protein interface formation, with higher values meaning higher probability. A PIER index above 30 indicates a likely protein-protein binding interface residue, and below zero an unlikely interface residue.

ConSurf calculates a conservation score for each protein residue based on a PSI-BLAST alignment of unique homologous sequences found in UniProtKB/Swiss-Prot [66]. For each protein residue, ConSurf provides a normalized score, so that the average score for all residues in the protein is zero, and the standard deviation is one. The conservation scores calculated by ConSurf are a relative measure of the evolutionary conservation at each residue of the target protein. The lowest scores represent the most conserved positions in the protein. ConSurf provides output data in different formats. In this analysis the "Amino Acid Conservation Score" output files were used. These files provide, together with normalized conservation score for each residue, residue color values (scale of 1–9) and confidence intervals for the conservation score and color (for the Bayesian method of calculation which is used by default). Amino acid positions that are assigned confidence intervals that are too large to be trustworthy are marked in the output files. Both all residues with conservation scores and residues for which scores were confident (not marked in the output files of the ConSurf server), i.e., a confident interval assigned to the score was less than 50% [56], were used in this study.

ClusPro running the DOT program returns the ten best models as one PDB formatted file re-numerating protein chains, residues and atoms. DOT is limited to proteins not exceeding 3,700 atoms.

PatchDock returns up to 2,000 models each as a separate PDB formatted file and provides the option to retain the 100 best models in one archive file. The ten best (by model score) were used in the current analysis. Also the filter for antigen-antibody interactions provided by PatchDock was used. That is, surface patches intersecting the CDR regions of the antibody. CDRs are detected by aligning the sequence of the given antibody to a consensus sequence from a library of antibodies [64].

ClusPro and PatchDock differ from the other servers tested by providing protein-protein docking. To use these servers the user needs to provide the structure of the antibody along with the antigen structure. We used the structures of protein antigen and antibody from the same complex, hence, only bound docking was considered. As was shown by the authors of the method, PatchDock bound docking substantially out-performed unbound docking [64].

The AUC values for scale-based methods were calculated using the algorithm of Tom Fawcett [61]. For discrete classifiers, that is, methods producing the only point on the ROC plot with coordinates {*x*; *y*}, the AUC was calculating as 0.5 * (1 - *x *+ *y*).

Molecule images were produced using the WebLabViewer software (Accelrys Inc.).

## Abbreviations

CDR – Complementary Determined Region of the Antibody.

Fab – antigen-binding fragment of antibody that includes one complete light chain paired with one heavy chain fragment containing the variable domain and the first constant domain.

VHH – antigen-binding fragment of the antibody that includes the variable domain of the heavy chain.

Fv – antigen-binding fragment of antibody that includes variable domains of heavy and light chains.

scFv – antigen-binding fragment of the antibody that includes the covalently linked variable domains of the heavy and light chains.

TCR – T Cell receptor.

x¯
 MathType@MTEF@5@5@+=feaafiart1ev1aaatCvAUfKttLearuWrP9MDH5MBPbIqV92AaeXatLxBI9gBaebbnrfifHhDYfgasaacH8akY=wiFfYdH8Gipec8Eeeu0xXdbba9frFj0=OqFfea0dXdd9vqai=hGuQ8kuc9pgc9s8qqaq=dirpe0xb9q8qiLsFr0=vr0=vr0dc8meaabaqaciaacaGaaeqabaqabeGadaaakeaaieaacuWF4baEgaqeaaaa@2E42@ – sample arithmetic mean.

*s *– sample standard deviation.

TP, FP, TN, FN – true positives, false positives, true negatives, and false negatives, respectively.

ROC – Receiver Operating Characteristics.

AUC – area under the ROC curve.

## Authors' contributions

JVP conceived, designed and performed the research including data collection and analysis. PEB suggested extensions and modifications to the research. Both JVP and PEB wrote the manuscript. The authors have read and approved the final version of the manuscript.

## Supplementary Material

Additional file 1 The representative structures of protein antigens (numbered) and antibody-protein complexes represented different epitopes for each antigen (epitopes inferred from one-chain antibody fragments are in italic). The data provides curated information on 82 3D structures of antibody-protein complexes (dataset #1) represented 169 structures of antibody-protein complexes available in the PDB of January, 2006 and used in this work. Click here for file

Additional file 2 The detailed statistics on the prediction results for 59 representative epitope. This table provides additional information that complements the Tables 2 and 3. The analysis was performed using 59 representative epitopes from dataset #2 that were inferred from structures of one-chain (monomer) antigens in complexes with two-chain antibody fragments. Click here for file
