# Supplementary material for: Antibody-protein interactions: benchmark datasets and prediction tools evaluation
Source: BMC Struct Biol. 2007 Oct 2;7:64. doi: 10.1186/1472-6807-7-64 (PMC2174481; doi:10.1186/1472-6807-7-64)
Supplement: Additional file 1 — The representative structures of protein antigens (numbered) and antibody-protein complexes represented different epitopes for each antigen (epitopes inferred from one-chain antibody fragments are in italic). The data provides curated information on 82 3D structures of antibody-protein complexes (dataset #1) represented 169 structures of antibody-protein complexes available in the PDB of January, 2006 and used in this work. [file 1472-6807-7-64-S1.doc]

**Supplemental Table 1.** The representative structures of protein antigens (numbered) and antibody-protein complexes represented different epitopes for each antigen (epitopes inferred from one-chain antibody fragments are in italic)

| N | PDB ID | Res,  Å | Antigen Chains Interacting with Antibody | Antigen Name, chain/domain name interacting with antibody | Antigen Specie | Antibody Name | Antibody Fragment | Antibody Specie | Heavy Chain Isotype | Heavy Chain PDB ID | Light Chain Isotype | Light Chain PDB ID |
| --- | --- | --- | --- | --- | --- | --- | --- | --- | --- | --- | --- | --- |
| 1 | 1AFV | 3.7 | A | capsid protein p24 N-terminal domain (homodimer), monomer | HIV-1 | 25.3 | Fab | M. musculus | IgG1 | H | κ | L |
| 2 | 1BGX | 2.3 | T | Taq DNA polymerase I | Thermus aquaticus | inhibitory TP7 | Fab | M. musculus | IgG2a | H | κ | L |
| *3* | *1BZQ* | *2.8* | *A* | *RNAse A* | *Bos taurus* | *cAb-RN05* | *VHH* | *C. dromedarius* | *Ig* | *L* |  |  |
| 4 | 1E6J | 3 | P | capsid protein p24 243 residue recombinant variant RH24 monomer, C-terminal domain | HIV-1 strain HXB2 | 13B5 | Fab | M. musculus | IgG1 | H | κ | L |
| 5 | 1EGJ | 2.8 | A | cytokine GM-CSF/IL-3/IL-5 receptor, β-chain signaling subunit, activation domain | H. sapiens | BION-1 | Fab | M. musculus | IgG2a | H | κ | L |
| 6 | 1FSK | 2.9 | A | major birch pollen allergen Bet v 1-A | Betula pendula | BV16 | Fab | M. musculus | IgG1 | C | κ | B |
| *7* | *1G6V* | *3.5* | *A* | *erythrocyte carbonic anhydrase* | *Bos taurus* | *cAb-CA05* | *VHH* | *C. dromedarius* | *Ig* | *K* |  |  |
| 8 | 1H0D | 2 | C | angiogenin | H. sapiens | 26-2F | Fab | M. musculus | IgG | B | κ | A |
| 9 | 1I9R | 3.1 | A | CD40L globular part of extracellular domain (homotrimer), monomer | H. sapiens | humanized murine 5c8 | Fab | H. sapiens | IgG1 | H | κ | L |
| 10 | 1IAI | 2.9 | I, M | mouse mAb IgG2a 730.1.4. Fab | M. musculus | 409.5.3 | Fab | M. musculus | IgG1 | H | κ | L |
| 11 | 1IQD | 2 | C | factor VIII, C2 domain | H. sapiens | BO2C11 | Fab | H. sapiens | IgG4 | B | κ | A |
| 12 | 1JRH | 2.8 | I | IFNγR, α-chain, N-terminal domain, mutation C105S | H. sapiens | A6 | Fab | M. musculus | IgG1 | H | κ | L |
| 13 | 1KB5 | 2.5 | A,B | T cell receptor KB5-C20, heterodimer | M. musculus | Desire-1 | Fab | M. musculus | IgG2a | H | κ | L |
| 14 | 1LK3 | 1.91 | A | cytokine interleukin 10, IL-10, monomer | H. sapiens | 9D7 | Fab | R. norvegicus | IgG1 | H | κ | L |
| 15 | 1MHP | 2.8 | B | α1β1 integrin, domain I | chimeric | huAQC2 | Fab | H. sapiens | IgG1 | X | κ | Y |
| *16* | *1MVF* | *1.65* | *E,D* | *labil antidote MazE (homodimer)* | *E.Coli* | *cAbMaz1* | *VHH* | *C. dromedarius* | *Ig* | *A* |  |  |
| 17 | 1NL0 | 2.2 | G | factor IX, calcium-stabilized Gla domain | H. sapiens | 10C12 | Fab | H. sapiens | IgG1 | H | λ | L |
| 18 | 1NSN | 2.9 | S | Staphylococcal nuclease (Snase) | S. aureus | N10 | Fab | M. musculus | IgG1 | H | κ | L |
| 19 | 1OAZ | 2.77 | A | Trx-Shear3, recombinant thioredoxin | synthetic | SPE7 | Fv | R. rattus | IgE | H | λ | L |
| *20* | *1OP9* | *1.86* | *B* | *lysozyme* | *H. sapiens* | *cAb-HuL6* | *VHH* | *C. dromedarius* | *Ig* | *A* |  |  |
| 21 | 1ORQ | 3.2 | C | KvAP K(+) potassium channel (homo-tetramer), monomer | Aeropyrum pernix | 6E1 | Fab | M. musculus | IgG2a | B | κ | A |
| 22 | 1ORS | 1.9 | C | KvAP K(+) potassium channel (homo-tetramer), monomer | Aeropyrum pernix | 33H1 | Fab | M. musculus | IgG1 | B | κ | A |
| 23 | 1PG7 | 2.5 | H,L | humanized anti-tissue factor D3H44 IgG1/κ antibody, Fab, heterodimer | H. sapiens | 6A6 | Fab | M. musculus | IgG2a | X | λ | W |
| 24 | 1PKQ | 3 | E | myelin oligodendrocyte glycoprotein, extracellular domain | R. rattus | 8-18C5 | Fab | M. musculus | IgG1 | B | κ | A |
| 25 | 1RJL | 2.6 | C | outer surface protein B (OspB), C-terminal fragment (residues 152-296) | Borrelia burgdorferi | bactericidal H6831 | Fab | M. musculus | IgG2a | B | κ | A |
| 26 | 1RVF | 4 | 1,2,3 | 14 coat protein hetero-tetramer | Human rhinovirus 14 | 17-IA | Fab | M. musculus | Ig | H | κ | L |
| 27 | 1SY6 | 2.1 | A | T cell receptor CD3-ε/γ ectodomain, heterodimer fused in one chain | H. sapiens | OCT3 | Fab | M. musculus | IgG2a | H | κ | L |
| 28 | 1TZI | 2.8 | V | hVEGF (homodimer), monomer | H. sapiens | YADS2 | Fab | M. musculus | IgG1 | B | κ | A |
| 29 | 1WEJ | 1.8 | F | cytochrome c | Equus caballus | E8 | Fab | M. musculus | IgG1 | H | κ | L |
| 30 | 1YJD | 2.7 | C | CD28 extracellular domain (homodimer), monomer | H. sapiens | 5.11A1 | Fab | M. musculus | IgG1 | H | κ | L |
| 31 | 1YNT | 3.1 | F | major surface antigen SAG1 | Toxoplasma gondii | 4F11E12 | Fab | M. musculus | IgG2a | B | κ | A |
| 32 | 1YY9 | 2.6 | A | EGFR, extracellular region | H. sapiens | cetuximab (Erbitux) | Fab | H. sapiens | IgG1 | D | κ | C |
| 33 | 1ZA3 | 3.35 | R | death receptor 5, extracellular domain | H. sapiens | anti-hVEGF-YSd1 | Fab | H. sapiens | IgG1 | H | κ | L |
| 34 | 1ZTX | 2.5 | E | envelope protein, domain III | West Nile virus | E16 | Fab | M. musculus | IgG2b | H | κ | L |
| 35 | 2JEL | 2.5 | P | histidine-containing protein HPr | E. coli | Jel42 | Fab | M. musculus | IgG1 | H | κ | L |
| 36 | 1A14 | 2.5 | N | neuraminidase | Influenza a virus | recombinant NC10 (scFv(5)) | scFv | M. musculus | Ig | H | κ | L |
|  | 1NCA | 2.5 | N | neuraminidase | Influenza A virus (H11N9) | NC41 | Fab | M. musculus | IgG2a | H | κ | L |
| *37* | *1RJC* | *1.4* | *B* | *HEL, hen egg white lysozyme* | *Gallus gallus* | *cAb-Lys2* | *VHH* | *C. dromedarius* | *Ig* | *A* |  |  |
|  | 1A2Y | 1.5 | C | HEL, hen egg white lysozyme | Gallus gallus | D1.3 | Fv | M. musculus | Ig | B | κ | A |
|  | *1DZB* | *2* | *X* | *TEL, turkey egg lysozyme* | *Meleagris gallopavo* | *1F9* | *scFv* | *M. musculus* | *Ig* | *A* |  |  |
|  | 1JHL | 2.4 | A | lysozyme | Ring-necked pheasant | D11.15 anti-HEL | Fv | M. musculus | IgG1 | H | κ | L |
|  | *1JTO* | *2.5* | *L* | *HEL, hen egg white lysozyme* | *Gallus gallus* | *cAb-Lys3* | *VHH* | *C. dromedarius* | *Ig* | *A* |  |  |
|  | 1NDG | 1.9 | C | HEL, hen egg white lysozyme | Gallus gallus | HyHEL-8 | Fab | M. musculus | IgG2a | B | κ | A |
|  | 1P2C | 2 | C | HEL, hen egg white lysozyme | Gallus gallus | F10.6.6 | Fab | M. musculus | IgG1 | B | κ | A |
|  | *1RI8* | *1.85* | *B* | *HEL, hen egg white lysozyme* | *Gallus gallus* | *D2-L19* | *VHH* | *C. dromedarius* | *Ig* | *A* |  |  |
| 38 | 1JPS | 1.85 | T | tissue factor (TF), extracellular domain | H. sapiens | humanized D3h44 | Fab | H. sapiens | IgG1 | H | κ | L |
| 39 | 1AR1 | 2.7 | B | cytochrome c oxidase core subunits I and II (heterodimer), subunit II | Paracoccus denitrificans | 7E2C50S | Fv | M. musculus | Ig | C | κ | D |
| 40 | 1BJ1 | 2.4 | V, W | hVEGF, homodimer | H. sapiens | A4.6.1 Fab-12 | Fab | M. musculus | Ig | K | κ | J |
|  | 1CZ8 | 2.4 | V, W | hVEGF, homodimer | H. sapiens | In-vitro matured A4.6.1 Fab-12 (Y0317) | Fab | M. musculus | Ig | H | κ | L |
|  | 1TZH | 2.6 | W, V | hVEGF, homodimer | H. sapiens | YADS1 | Fab | M. musculus | IgG1 | H | κ | L |
| 41 | 1EO8 | 2.8 | A | hemagglutinin (HA1 chain and HA2 fragment), HA1 chain | Influenza A virus (X31) | BH151 | Fab | M. musculus | IgG1 | H | κ | L |
|  | 1KEN | 3.5 | A | hemagglutinin (trimer), HA1 chain | Influenza A virus (X31) | HC63 | Fab | M. musculus | IgG1 | H | λ | L |
|  | 1QFU | 2.8 | A | hemagglutinin (HA1 chain and HA2 fragment), HA1 chain | Influenza A virus (X31) | HC45 | Fab | M. musculus | IgG1 | H | κ | L |
|  | 2VIT | 3.25 | C | hemagglutinin HA1 chain monomer, escape mutant T155I | Influenza A virus (X31) | HC19 | Fab | M. musculus | IgG1 | B | λ | A |
| 42 | 1EZV | 2.3 | E | cytochrome bc(1) (hetero-octadecamer), Rieske protein monomer | S. cerevisiae | 18E11 | Fv | M. musculus | Ig | X | κ | Y |
| 43 | 2ADF | 1.9 | A | von Willebrand factor, A3 domain | H. sapiens | 82D6A3 | Fab | M. musculus | IgG2a | H | κ | L |
|  | 1FE8 | 2.03 | A | von Willebrand factor, A3 domain | H. sapiens | RU5 | Fab | M. musculus | IgG2a | H | κ | L |
| 44 | 1OSP | 1.95 | O | outer surface protein A (OspA) | Borrelia burgdorferi | 184.1 | Fab | M. musculus | IgG2b | H | κ | L |
|  | 1FJ1 | 2.68 | F | outer surface protein A (OspA) | Borrelia burgdorferi | LA-2 | Fab | M. musculus | IgG2b | B | κ | A |
| 45 | 1FNS | 2 | A | von Willebrand factor, A1 domain | H. sapiens | NMC-4 | Fab | M. musculus | IgG1 | H | κ | L |
| 46 | 1G9M | 2.2 | G | gp120 envelope glycoprotein | HIV-1 strain HXBc2 | 17B | Fab | H. sapience | IgG1 | H | κ | L |
|  | 2B4C | 3.3 | G | gp120 core with V3 region | HIV-1 | X5 | Fab | H. sapiens | IgG1 | H | κ | L |
| 47 | 1R0A | 2.8 | B | reverse transcriptase p66/p51 heterodimer, p66 chain | HIV-1 | Fab28 | Fab | M. musculus | IgG1 | H | κ | L |
| 48 | 1R3J | 1.9 | C | KcsA K+ voltage-gated potassium channel (homo-tetramer), monomer | Streptomyces coelicolor | unnamed antibody | Fab | M. musculus | IgG1 | B | κ | A |
| *49* | *1KXQ* | *1.6* | *A* | *porcine pancreatic alpha-amilase* | *Sus scrofa* | *AMD9 VHH* | *VHH* | *C. dromedarius* | *Ig* | *H* |  |  |
|  | *1KXT* | *2* | *A* | *porcine pancreatic alpha-amilase* | *Sus scrofa* | *AMB7 VHH* | *VHH* | *C. dromedarius* | *Ig* | *B* |  |  |
|  | *1KXV* | *1.6* | *A* | *porcine pancreatic alpha-amilase* | *Sus scrofa* | *AMD10 VHH* | *VHH* | *C. dromedarius* | *Ig* | *C* |  |  |
| 50 | 1N8Z | 2.52 | C | epidermal growth factor receptor 2 (HER2, Neu, ErbB2), extracellular region | H. sapiens | Herceptin (trastuzumab) | Fab | H. sapiens | IgG1 | B | κ | A |
|  | 1S78 | 3.25 | B | epidermal growth factor receptor 2 (HER2, Neu, ErbB2), extracellular region | H. sapiens | pertuzumab (2C4,Omnitarg) | Fab | M. musculus | IgG1 | F | κ | E |
| 51 | 1NFD | 2.8 | D | T cell receptor αβ N15 heterodimer, β-chain | M. musculus | anti-TCR H57 | Fab | Cricetinae gen. sp. | Ig | H | λ | G |
| 52 | 1OB1 | 2.9 | C | surface protein 1 (monomer) | Plasmodium falciparum | G17.12 | Fab | M. musculus | IgG2a | B | κ | A |
| 53 | 1OTS | 2.51 | A,B | ClC chloride channel, homodimer | E.coli | unnamed antibody | Fab | M. musculus | IgG1 | C | κ | D |
| 54 | 1QFW | 3.5 | A,B | chorionic gonadotropin hCG heterodimer | H. sapiens | anti-α-hCG 3299 | Fv | M. musculus | Ig | H | κ | L |
|  | 1QFW | 3.5 | A,B | chorionic gonadotropin hCG heterodimer | H. sapiens | anti-β-hCG 3468 | Fv | M. musculus | Ig | I | κ | M |
| 55 | 1TQB | 2.55 | A | prion protein, native VRQ variant, C-terminal domain | Ovis aries | VRQ14 | Fab | M. musculus | IgG2a | B | κ | C |
| 56 | 1TXV | 2.75 | A | platelet integrin α(IIb)β3 (heterodimer), integrin α(IIb) | H. sapiens | E6 | Fab | M. musculus | IgG2a | H | κ | L |
| 57 | 1V7M | 2.51 | V | cytokine thrombopoietin (hTPO), N-terminal receptor-binding domain | H. sapiens | TN1-neutralizing IgG1 | Fab | M. musculus | IgG1 | H | κ | L |
| 58 | 1XIW | 1.9 | A | T cell receptor CD3-ε/δ ectodomain heterodimer, ε chain | H. sapiens | UCHT1-scFv | scFv | not specified | Ig | D | κ | C |
| 59 | 1XIW | 1.9 | F | T cell receptor CD3-ε/δ ectodomain heterodimer, δ chain | H. sapiens | UCHT1-scFv | scFv | not specified | Ig | D | κ | C |
| 60 | 1Z3G | 3.3 | A | surface protein P25 | Plasmodium reichenowi | 2A8 | Fab | M. musculus | IgG1 | H | κ | L |
| 61 | 2AEP | 2.1 | A | Neuraminidase monomer | influenza virus (H3N2) | Mem5 | Fab | M. musculus | IgG2b | H | κ | L |
| *62* | *2BSE* | *2.7* | *B, C* | *receptor-binding protein, homotrimer* | *L. lactis bacteriophage p2* | *VHH5* | *VHH* | *Lama glama* | *Ig* | *D* |  |  |
